# Human Toxicity and Marine Ecotoxicological Risk of Methanol as a Ship Fuel: A Critical Narrative Review of Exposure, Fate and Aquatic Effects

**DOI:** 10.3390/toxics14100868

**Published:** 2026-09-29

**Authors:** Dimitrios Parris, Stavros Kalogiannidis, Konstantinos Spinthiropoulos, Fotios Chatzitheodoridis

**Affiliations:** 1 Department of Management Science and Technology, University of Western Macedonia, Koila, 50100 Kozani, Greece; kspinthiropoulos@uowm.gr (K.S.); fxtheodoridis@uowm.gr (F.C.); 2 Department of Business Administration, University of Western Macedonia, 51100 Grevena, Greece

**Keywords:** methanol, marine fuel, formate toxicity, occupational exposure limits, environmental fate, aquatic ecotoxicology, bunkering safety, chemical risk assessment, evidence provenance

## Abstract

Methanol is being adopted as a marine fuel on a safety profile that is quoted more often than it is checked. This review audits the provenance of that profile. Four load-bearing claims—rapid dilution, a one-to-seven-day aqueous half-life, negligible bioaccumulation and manageable human toxicity—were traced backward from the maritime literature to the document generating each, every transfer was examined for whether the measurement conditions stated at origin were carried forward, and every record was verified against the issuing body’s own version. Of nine quantitative claims traced, two reached the sector with their conditions intact, five lost them, and two rest on primary records that could not be retrieved, with one being the only marine acute determination located. Three mechanisms recur: one term, half-life, carrying both a clinical elimination window and an environmental turnover figure; values transmitted without the method that produced them; and one property cited for opposite conclusions by bodies that do not cite each other. Methanol remains milder than heavy fuel oil and ammonia, but conditionally so. The first gas alarm, at twenty percent of the lower explosion limit, falls near 11,000 to 12,000 ppm, whereas 6000 ppm is immediately dangerous to life.

## 1. Introduction

Methanol has moved from candidate to commitment. Fifty-nine methanol dual-fuel ships were in service and 369 on order as of May 2025 [1], and the reasons are not in dispute: the fuel is liquid at ambient temperature and pressure, requires no cryogenic containment, and delivers immediate reductions in sulfur oxides and particulate matter against residual fuel oil [2,3]. The comparative case has been made repeatedly, including in an earlier synthesis by the present authors [2], and nothing in this review disturbs it. On persistence, bioaccumulation and acute aquatic toxicity, methanol remains the milder option among the leading alternative fuels.

That verdict rests on a small number of quantitative propositions that recur across classification guidance, response literature and peer-reviewed assessment: that a release dissolves and disperses below one percent within an hour of an open-sea spill; that methanol biodegrades in surface water with a half-life of one to seven days; that it does not bioaccumulate; and that its human toxicity, though severe, is a clinical problem with an established antidote. Each proposition is defensible in the setting that generated it. The question asked here is narrower, and prior to the comparative one: whether these propositions survive being traced to the documents that first stated them, and whether the conditions attached at origin have traveled with the numbers.

There is a specific reason to ask about this fuel. Methanol’s hazard is not legible from its property table. The clinical syndrome is not methanol syndrome but a formate syndrome, manufactured by the exposed organism itself and gated by a folate-dependent clearance pathway [4]; the latency between exposure and visual loss runs to tens of hours, so a shipboard exposure can be complete before it is recognized [5]. The same clearance capacity has been proposed as the explanation for the distribution of sensitivity across aquatic taxa [6], which means the familiar contrast between a substance that blinds humans and one aquatic testing classifies as practically harmless is not a contradiction between two toxicologies but one toxicology observed in organisms of differing metabolic competence at concentrations differing by orders of magnitude. A hazard of that shape is poorly served by documents carrying one number per property.

Three patterns recur across the corpus and are announced here because they, rather than any individual disagreement, constitute the finding of this review.

The first is a saturation of vocabulary. The term half-life carries at least two incompatible meanings in this literature. Clinically, methanol elimination is saturable, and reported half-lives span roughly 2.5 to 87 h because the value depends on the concentration at which it was measured and on whether alcohol dehydrogenase was pharmacologically blocked [4]. Environmentally, the one-to-seven-day figure is consistent with microbial turnover measured at ambient nanomolar concentrations, close to a half-saturation constant of 9.3 nM [7], at spill concentrations, six to eight orders of magnitude higher, the same kinetics are zero-order and the rate does not scale with load. Two saturation-limited quantities, both reported as half-lives, neither transferable beyond its own regime—and the shared word conceals the discontinuity.

The second is transfer without the method. A value measured under stated conditions is transcribed into a compilation that omits those conditions and is then quoted downstream as a method-independent property of the substance. Its mildest form is the flash point, given as 10 °C by the intergovernmental screening assessment, as typically 11 °C by ISO 6583:2024 and as 12 °C in the industry technical series [8,9].

**Figure 1:**
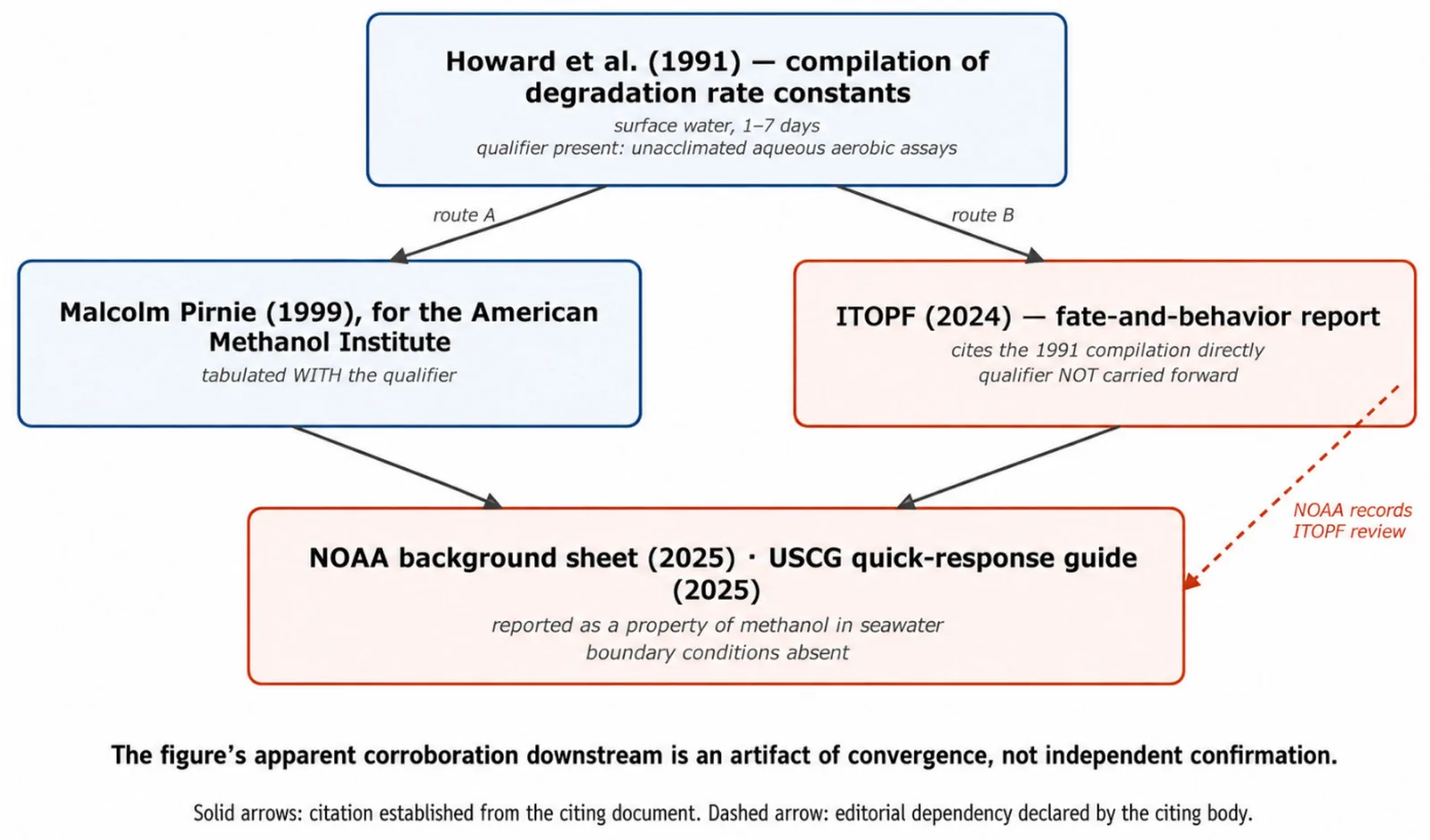
Transmission of the one-to-seven-day aqueous half-life from its origin to the 2025 response literature. The value is generated once, in unacclimated aqueous aerobic assays compiled in 1991, and reaches the response documents by two routes rather than one. The qualifier survives the first route and is shed on the second, and the two converge in documents that are themselves not independent, with the citing body recording editorial review by the transmitting one. This is what the review calls pseudo-convergence: several documents appear to confirm a value independently when, in fact, all of them descend from a single original measurement, so that repetition is read as replication. The apparent weight of evidence is then an artifact of counting one determination more than once. Boxes outlined in blue denote documents in which the measurement qualifier is present; boxes outlined in red denote documents in which it has been lost. Solid arrows: citation established from the citing document. Dashed arrow: editorial dependency declared by the citing body. Full citations for each document appear in Section 1, Section 5.3 and Section 9.1 [8,10,11,12,14].

The spread has no operational consequence and is recorded only because the mechanism recurs at a greater magnitude. Its consequential form is the aqueous half-life, whose chain can be reconstructed link by link: unacclimated aqueous aerobic assays were compiled in a 1991 handbook of degradation rates [9,10], tabulated with that qualifier in a 1999 consultancy assessment [11], taken up independently from the same compilation by the industry technical report [8], and reproduced by federal response agencies in 2025 as a property of methanol in seawater [8,12,13,14]. Figure 1 sets out the chain in outline; Section 5 reconstructs it link by link and identifies the transfer at which the qualifier disappears.

The third is one property, opposite conclusions. Complete miscibility is used in a 2026 combustion study to argue that spills dilute rapidly and environmental harm is thereby minimized [15] and, in the same year, by a response guide to argue that methanol can be neither boomed, skimmed nor absorbed, so that releases into enclosed or low-energy waters may affect water quality and local biota [8,13,14]; neither document cites the other. The low Henry’s law constant establishes, in the words of the assessing body itself, that volatilization is not a significant removal process from the aquatic compartment, and the same low value is invoked within the same document to justify accepting acute aquatic toxicity results obtained in unsealed vessels—so that retention in the water column is filed as evidence of environmental safety [9].

The consequences are not confined to the literature. The interim guidelines for ships using methyl or ethyl alcohol as a fuel set their detection logic against the lower explosion limit, with an alarm at twenty percent and the activation of the safety system at forty percent [16]. With a lower explosion limit reported between 55,000 and 60,000 ppm, the first alarm falls between roughly 11,000 and 12,000 ppm, whereas the concentration immediately dangerous to life or health is 6000 ppm and the eight-hour AEGL-3 is 1600 ppm [3,17]. The ammonia guidelines issued five years later, on the same goal-based template, set their triggers in parts per million at 25, 110 and 220 [18]. Concentration-anchored triggers are therefore available and have simply not been drafted for methanol. Computational work meanwhile puts a machinery-space vapor cloud at 2.95 m within seconds of a large-bore release, above standing breathing height, with no toxicological threshold applied to that field [19].

This review is accordingly an audit of provenance, not a re-derivation of hazard. It generates no new data and does not claim to perform the operations it finds missing: where no located source aggregates dermal and inhalational exposure into one body burden or derives a predicted no-effect concentration for seawater, the deficiency is recorded as a gap. Section 2 states the method and the selection rule. Section 3, Section 4, Section 5, Section 6, Section 7 and Section 8 take in turn the property set, the human toxicology and the disharmony of occupational limits, environmental fate, exposure scenarios from bunkering to accidental release, ecotoxicological effects on aquatic organisms, and the regulatory architecture in which toxicity falls between three instrument families that each describe a different methanol. Section 9 and Section 10 draw the discussion and conclusions together. Eight research gaps emerge from the audit and are set out and ranked in Section 9.3: marine-specific fate and transport data; water toxicity following an incident; environmental assessment criteria for the marine leakage of alternative fuels; the aggregation of dermal, inhalational and ocular exposure into a single shipboard dose; bunkering frequency expressed as exposure per crew role; an integrated matrix of volumetric energy density, occupational limits and marine endpoints; the unreconciled energy-density series; and the incident record itself, which is assembled largely outside the maritime reporting system. Section 9.3 ranks the first, second and fourth of these as first-order, together with the absence of acute and chronic determinations generated in seawater to a current test guideline, and names the experimental and modeling work that should be funded first. They are established there rather than here because a gap is claimed only where a published source declares it or the absence is demonstrable from the corpus assembled under Section 2.3, and neither condition can be tested before that corpus exists. One rule governs throughout: every analytical distinction deployed is attributed to a published source that draws it explicitly, and the criticism is directed at the non-application of distinctions that already exist.

## 2. Methods and Scope

### 2.1. Review Type and the Reason for It

This article is a critical narrative review. The claims examined lie across four literatures that do not share a vocabulary: clinical and occupational toxicology, marine microbiology and biogeochemistry, maritime engineering and risk assessment; and the normative and gray literature of standards bodies, classification societies, protection and indemnity clubs and response agencies. Systematic and narrative reviews serve different purposes, with the first summarizing data on a narrow question and the second interpreting and criticizing heterogeneous work [20,21].

The question pursued here—whether the quantitative claims underpinning the current appraisal of methanol survive being traced to their origins—belongs to the second. It is also a review of complex evidence, for which the audited experience is that protocol-driven searching recovers roughly thirty percent of relevant material while pursuing references of references recovers about half [22]. A value transcribed without its test method, or a half-life without the concentration regime in which the underlying rate was measured, is joined to its origin by transmission rather than by shared terms and is reachable only backwards along the citation. Conventions of the systematic form that remain applicable—explicit selection, a stated and uniformly applied treatment of source currency, disclosure of unverified records—are adopted [23]. The limitations of the narrative form are stated in Section 9.5.

### 2.2. Purposive Selection: The Claim as Unit of Analysis

The unit of analysis is the claim, not the study. A document was included where it performs one of three functions in respect of a claim under examination: it originates the claim, reporting the primary measurement or derivation; it transmits it, in a compilation, guidance note, review or fact sheet; or it adjudicates it, as an assessment, standard or instrument pronouncing on its adequacy. The functions are not ranked: a transmitting document is admitted precisely because it transmits, and its weight here lies in what it does to the claim rather than in its own authority.

Two consequences follow. The selection is not a sample from which prevalence can be inferred, so the recurrence of a figure is evidence of propagation and not of corroboration; and inclusion is not endorsement. Because the corpus is assembled around the claims under examination rather than sampled from the literature, the patterns set out in Section 9 are demonstrated in the chains reconstructed here and are not offered as estimates of their prevalence in the wider field.

### 2.3. Retrieval and Verification

Sources were identified through Scopus, Web of Science, PubMed and Google Scholar, through direct retrieval from the issuing bodies (IMO, ISO, OECD, WHO/IPCS, GESAMP, NOAA, the USCG Research and Development Center, ITOPF, classification societies), and by backward citation chasing from every document carrying a quantitative claim examined in Section 3, Section 4, Section 5, Section 6, Section 7 and Section 8. Searching ended on 16 August 2026.

The corpus retained for analysis comprises the documents listed in the references, of which rather more than four-fifths are bibliographic sources and the remainder normative instruments or methodological references under the test set out in Section 2.4. Each record was verified against the publisher’s or the issuing body’s own record of the item; reference lists held by aggregators, subject indexes and repositories were not treated as authority for authorship, title or pagination—a precaution reinforced when one such list was found to carry an incorrect author attribution for an item already in the corpus.

No digital object identifier has been constructed or inferred; where a record remained incomplete after that check, no claim is allowed to rest on it except conditionally, and the dependence is stated at the point of use. Tracing did not uniformly find fault: the bioaccumulation and persistence propositions examined in Section 7.5 were traced to primary determinations that carry their conditions with them and are reported here as sound.

### 2.4. The Currency of Sources and the Status of Normative Instruments

The article prioritizes current evidence wherever currency bears on the claim but sets no numerical recency threshold. An audit of provenance rests of necessity on the older records in which the claims examined originate, so a target expressed as a proportion of recent items would penalize precisely the material the method requires. The resulting profile is nevertheless weighted toward current work: about three-fifths of the bibliographic sources were published within the five years preceding the end of searching, and the material predating 2010 amounts to some two dozen items, concentrated in the primary determinations and compilations from which the claims under examination descend.

An audit unable to reach a 1991 compilation of degradation rates, or a 1996 determination of acute toxicity in a marine bivalve, could not establish where a value originated, which is the question this review exists to answer. Two categories are excluded from that denominator on a functional test. Guidance on gray literature recommends that non-peer-reviewed material be classified by the function it performs and by the control exercised over its production, not by its publication type [24], and one strand of that guidance separates gray literature, which reports, from gray information, which directs [25].

The test applied here specializes that principle to a corpus in which some documents prescribe. An item counts as a bibliographic source when it reports data, an analysis or an argument and as a normative instrument when it prescribes a requirement, procedure, specification or limit value; instruments are excluded because their currency is set by force and amendment status rather than by date of issue.

Methodological references, cited only for the conduct of the review, are excluded on the same principle. The test resolves the three hybrid documents in this corpus: the OECD Screening Information Data Set assessment and the WHO Environmental Health Criteria monograph count as sources, with their function here being to report measured values, whereas the revised GESAMP hazard evaluation procedure and the acute exposure guideline documentation are treated as instruments, with their function being to prescribe endpoints, pass criteria and guideline values. The assignment holds uniformly across all sections. Material published before 2023 is admitted where primary, foundational or normative and is identified as such at the point of use. The classification of every entry in the reference list under this test is set out in Table S1 (Supplementary Materials).

### 2.5. Scope Boundaries and the Attribution Rule

Within scope are the human toxicology of methanol, the exposure pathways arising aboard ship and during bunkering, environmental fate in the aquatic compartment, ecotoxicological effects on aquatic organisms, and the occupational and emergency limit values governing them. Outside scope are well-to-wake greenhouse gas accounting, production pathways, techno-economics, engine performance and combustion emissions, except where these bear directly on exposure. No new experimental data are generated; arithmetic on published values, such as unit conversions and stoichiometric oxygen demand, is identified at the point of use.

**Figure 2:**
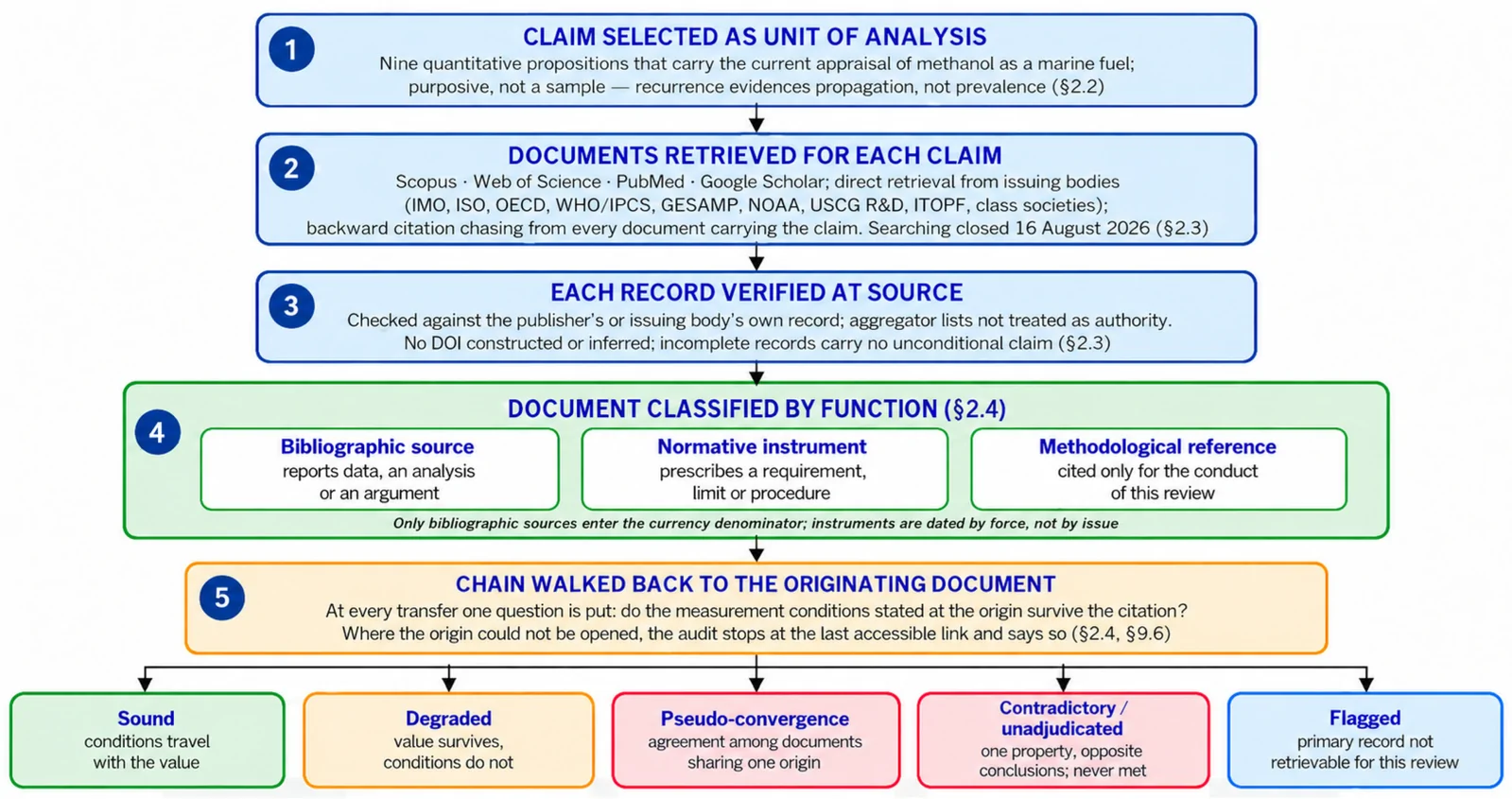
Procedure of the review. The unit of analysis is the claim rather than the study; documents enter the corpus by the function they perform in respect of a claim, and each chain is walked back to the document in which the value is generated. The test applied at every transfer is whether the measurement conditions stated at the origin survive the citation. The five outcomes shown are those recorded claim by claim in in the register present in Section 9.1.

One rule governs the critical apparatus. Every analytical distinction deployed here is attributed to a published source that draws it explicitly, and where a distinction exists in the literature but has not been carried into practice the criticism is directed at the non-application, not at an absence. Where no located source performs an operation the argument would require—the summation of dermal and inhalational contributions into one body burden, or the referencing of a modeled dispersion field against toxicological thresholds—the deficiency is recorded as a research gap and is not presented as work carried out here. The procedure set out in Section 2.2, Section 2.3, Section 2.4 and Section 2.5 is shown in outline in Figure 2.

### 2.6. Use of Generative AI Tools

**Figure 3:**
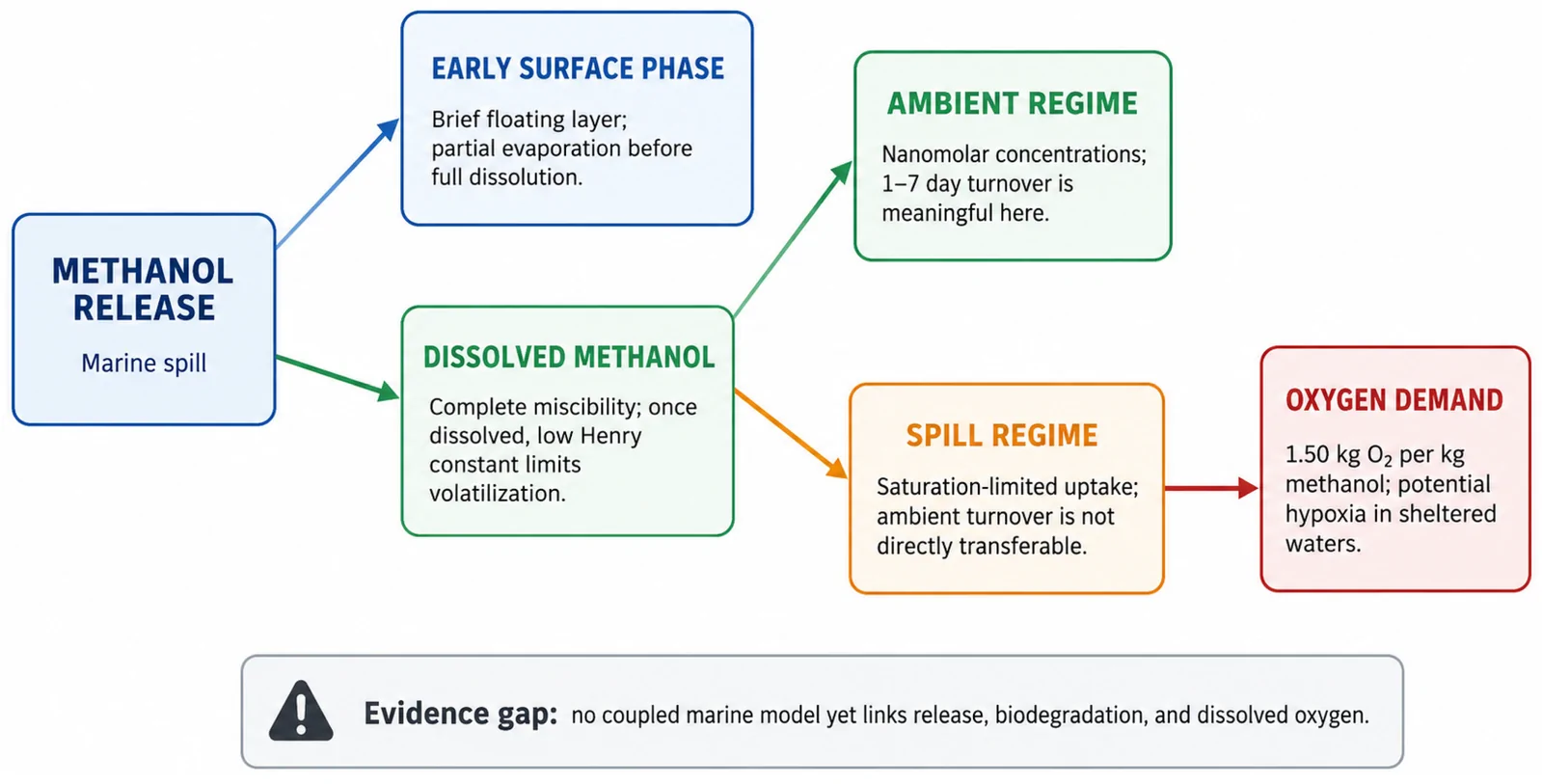
Phase-dependent environmental fate of marine methanol releases and the potential oxygen-demand pathway in sheltered waters.

**Figure 4:**
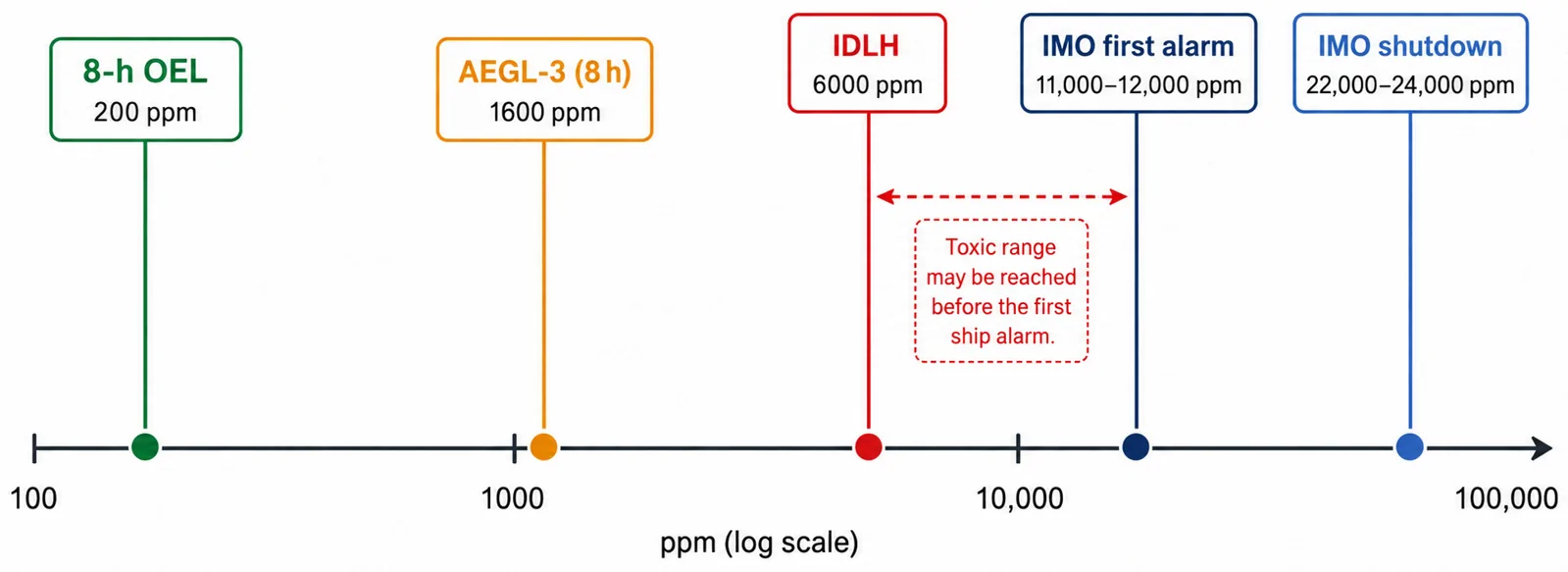
Methanol toxicological reference concentrations compared with IMO flammability-based detection thresholds. Markers in green, orange and red are toxicological reference concentrations; markers in blue are the IMO detection and shutdown thresholds, which are set by reference to the lower explosive limit.

A generative artificial intelligence tool, ChatGPT (OpenAI, GPT-5.6 Sol), was used at one stage of this work and is disclosed here in full. Figure 1, Figure 2, Figure 3 and Figure 4 and the graphical abstract were rendered and iteratively refined with the assistance of that tool; Figure 2 was added at revision and prepared on the same terms. In each case, the analytical content preceded the tool: the citation chains, the document sets and the relations depicted were established by the authors through the retrieval and verification procedure described in Section 2.3, specified by the authors in written form, and supplied to the tool as instructions for visual rendering only. Every rendered element was checked by the authors against the underlying analysis and against the source documents before inclusion and revised where the rendering did not correspond to them.

The tool was not used to identify, retrieve, select, verify, analyze or interpret any source, to generate or transform any quantitative value, or to draft the analytical text of this review. No output of the tool was accepted as evidence at any point. The authors reviewed and edited all AI-assisted output and take full responsibility for the content, accuracy and originality of the figures and of the manuscript as a whole.

## 3. Physicochemical Properties Governing Toxicity, Fate and Exposure

### 3.1. The Baseline Property Set and the Variance Already Present in It

Methanol enters the alternative-fuel debate with a property set that appears unusually secure. The peer-reviewed comparative appendix compiled by Lee and colleagues [3] gives a lower heating value of 19.9 MJ/kg, a volumetric energy density of 15.8 GJ/m^3^, a flash point of 11 °C, a flammable range of 6–36.5 vol % by volume in air, a vapor-to-air density ratio of 1.11 and complete miscibility with water. The screening assessment agreed under the OECD High Production Volume program at SIAM 19 [9], still the most comprehensive multi-endpoint compilation prepared for methanol under an intergovernmental process, adds a boiling point of 65 °C, a relative density of 0.79 g/cm^3^ and a vapor pressure of 127.9 hPa at 20 °C, and a closed-cup flash point of 10 °C.

The two flash points do not agree. A third value of 12 °C originates in the ITOPF alternative-fuels series [8] and is reproduced in the quick-response guidance of the United States Coast Guard Research and Development Center [12,13,14], while ISO 6583:2024 [26] warns users that methanol is a low-flash-point product with a flash point typically of 11 °C. The spread is two degrees, with no operational consequence at trading temperatures. It is recorded because the mechanism producing it recurs at greater magnitude later: a value measured under a stated method is transcribed into a secondary compilation without that method and then quoted as though it were method-independent. Where the property governs flammability, the drift is trivial; where it governs toxicity or fate, as Section 4 and Section 5 show, it is not.

### 3.2. The Henry Constant as the Hinge Between Two Incompatible Descriptions

The property doing most analytical work here is air–water partitioning. The OECD assessment reports a Henry law constant of 0.461 Pa·m^3^/mol at 25 °C, equivalent to 4.55 × 10^−6^ atm·m^3^/mol, and does not leave it open to interpretation: at this value, it states, volatilization is not a significant removal process from the aquatic compartment [9]. The reference compilation maintained by Sander (2023) [27] places methanol among the least strippable of the common industrial solvents; the conversion of the OECD value to the dimensionless gas-over-aqueous form gives approximately 1.9 × 10^−4^ at 25 °C, well below the range in which air stripping is treated as effective.

This sits uneasily beside the response literature, in which evaporation is routinely described as a dominant fate pathway. The reconciliation need not be constructed here: it has already been published, and turns on which phase is under discussion. Kass, Sluder and Kaul [13], writing for the United States Maritime Administration, describe a release onto open water as a buoyant surface layer that spreads and evaporates before it dissolves, with its dispersal into the water column depending on the mixing energy of waves and currents. The assessment prepared by Malcolm Pirnie for the American Methanol Institute [11] addresses the fraction already dissolved and reaches the opposite conclusion for precisely the reason the OECD assessment gives. Both hold within their own system; neither transfers to the other. What the operational literature does not consistently do is declare which system it means, and Section 5 documents the consequences for the half-life figures now in circulation.

A second use of the same constant appears inside the OECD assessment itself: in accepting acute aquatic toxicity results obtained in unsealed vessels, the report invokes the low Henry constant as evidence that nominal concentrations approximate measured ones [9]. The property thus establishes that methanol persists in the water column and simultaneously validates the test data from which the conclusion of low aquatic hazard is drawn. Both uses are internally consistent, yet together, they file retention in water under evidence of environmental safety.

### 3.3. Partitioning, Bioaccumulation, and the Oxygen Demand That No Framework Counts

The octanol–water partition coefficient is reported as a log Kow between −0.82 and −0.64, with measured bioconcentration factors below ten in Cyprinus carpio and Leuciscus idus and acute fish LC50 values of 15,400–29,400 mg/L in flow-through tests; the assessment concludes that methanol is of low priority for further environmental work owing to a low hazard profile [9]. Section 7 accepts that conclusion for direct toxicity and does not contest it.

The same molecular simplicity generates a second, separately quantified property. Complete oxidation consumes 1.5 mol of oxygen per mole, a theoretical oxygen demand of approximately 1.50 kg O_2_ per kilogram of fuel [11]. Saturated seawater carries of the order of 8 mg/L of dissolved oxygen, so one kilogram is stoichiometrically sufficient to strip the oxygen from roughly 190 cubic meters of receiving water. This is not a competing hazard estimate but the arithmetic consequence of the ready biodegradability on which the favorable assessment rests. The revised GESAMP hazard evaluation procedure [28] adopts oxygen consumption of at least sixty percent of the theoretical maximum within twenty-eight days as its pass criterion for ready biodegradability, and biochemical oxygen demand appears nowhere among its thirteen endpoints. The distinction between oxygen consumed as evidence of degradation and oxygen consumed as depletion of the receiving environment is internal to GESAMP’s own methodology; this review criticizes non-application, not absence.

The disagreement is live in the most recent literature. A dual-fuel combustion study published in 2026 [15] states that complete miscibility permits spills to dilute rapidly and thereby minimizes environmental harm, deriving the claim from the physical property alone. The Coast Guard guide [12,13,14], reasoning from the identical property, reaches the opposite conclusion: because methanol cannot be boomed, skimmed or absorbed, mechanical containment is impractical, and releases into enclosed or low-energy environments may produce short-term effects on water quality and local biota. It also records, as a stated knowledge gap, responder concern about water toxicity following methanol incidents. Miscibility is doing contradictory work in the two documents, and neither cites the other.

### 3.4. Vapor Density Above Unity and the Geometry of Enclosed Spaces

A vapor-to-air density ratio of 1.11 [3] is a modest excess, but it reverses the dispersion geometry assumed for the cryogenic alternatives. The computational fluid dynamics study of Sun and colleagues [19], modeling the engine room of a 249.8 m vessel as a compartment of 35 by 25 by 4 m, shows the cloud remaining at deck level precisely because its density exceeds that of air. A 12 cm breach fills the compartment within 15 s and produces a cloud 2.95 m high, 86 percent higher than for a 6 cm breach; raising the ambient temperature from 298–328 K extends the diffusion distance by 6.7 percent. Since 2.95 m exceeds standing breathing height, the property determines not only where the flammable envelope forms but where a person stands within it.

The descriptive literature is not unanimous even here. The ORNL spill study [13] states that methanol vapors rise and disperse rapidly, attributing this to high diffusivity in air. The NOAA background sheet [12], which cites that same study, describes off-gassed vapors as slightly denser than air and capable of traveling some distance over ground or water before dispersing; the ITOPF assessment reproduced in the Coast Guard guide [8,12,13,14] states that the vapor density prevents all but a small fraction of the cloud from rising, giving methanol a wider flammability footprint than the lighter alternatives. The measured density ratio and the computational result support the second description. Section 6 shows what follows when a model built on the first is used to size a safety zone.

### 3.5. Volumetric Energy Density and the Frequency of Transfer Operations

At 15.8 GJ/m^3^ against approximately 36 GJ/m^3^ for marine gas oil, methanol requires on the order of two to two and a half times the tank volume for equivalent range [3,29]. The consequence usually drawn is a design one. The operational consequence is that a given range bunkers more often, and each bunkering is a full cycle of connection, transfer, draining, purging and inerting, with every step being a discrete opportunity for release [17]. ISO 6583:2024 [26] specifies fuel quality at custody transfer and expressly assigns appropriate safety and health practices to the user, so operational frequency falls outside the quality standard.

Masum and colleagues [30] quantify the resulting trade-off between additional fuel volume and lost cargo capacity, but no source located for this review converts bunkering frequency into an expected annual exposure for a named crew role; the step from more frequent transfers to greater cumulative dose is therefore recorded as a research gap, not asserted as a finding.

### 3.6. Reading the Properties Side by Side

Table 1 assembles volumetric energy density and the available inhalation reference values for the candidate fuels from the peer-reviewed comparative appendix in [3] rather than from manufacturer safety data sheets, alongside an independent series compiled by the Society for Gas as a Marine Fuel [31] and reproduced in [12,13,14]. The two agree for methanol, ammonia and LNG but diverge sharply for hydrogen, where the choice between liquefied and compressed storage does work that neither table states. First, both the ammonia figure of 25 ppm and the methanol figure of 200 ppm were long in publication before either instrument was approved, yet only the ammonia value was written into one as a numerical trigger. Section 8 examines that asymmetry. Second, no publication located for this review assembles a matrix in which volumetric energy density, occupational exposure limits and marine environmental endpoints appear together for the same fuel set. That absence is stated as a gap in the literature, not as a deficiency this review has repaired, and it is the structural reason why the arguments of Section 4, Section 5, Section 6, Section 7 and Section 8 must be assembled from documents that do not cite one another.

## 4. Human Toxicology of Methanol

Methanol presents a paradox seldom stated explicitly: the parent molecule is among the least acutely potent of the low-molecular-weight alcohols, yet methanol poisoning is among the most feared alcohol exposures in emergency practice. Almost everything that makes methanol dangerous is manufactured by the exposed organism itself, a point on which recent syntheses converge [4,32,33]. This section reconstructs that transformation, identifies where the primary literature agrees and where it does not, and establishes the mechanistic vocabulary—formate as the operative toxicant—that Section 7 applies to aquatic organisms.

### 4.1. Toxicokinetics: The Formate Bottleneck and Its Interspecies Signature

Absorbed methanol distributes into total body water and is oxidized by hepatic alcohol dehydrogenase (ADH) to formaldehyde, which aldehyde dehydrogenase converts to formate within minutes.

Formaldehyde therefore never accumulates in vivo, and the clinical syndrome is entirely a formate syndrome. Formate inhibits mitochondrial cytochrome c oxidase (complex IV), producing histotoxic hypoxia in tissues with little oxidative reserve [34].

Two independent methodologies place the inhibition and clinical thresholds in close correspondence: kinetic work locates meaningful cytochrome oxidase inhibition above roughly 9–11 mmol/L serum formate [34,35], while outcome data from the Czech mass-poisoning cohort of thirty-eight patients associate serum formate at or above 11–12 mmol/L with visual and central nervous system sequelae and death [36].

That association is real but weaker than its downstream use suggests. Formate separated survivors without sequelae from the other outcome groups, but did not distinguish sequelae from death, nor one symptom pattern from another, and the area under the receiver operating characteristic curve for predicting survival without sequelae was 0.64, with a confidence interval from 0.44 to 0.85 that does not exclude chance [36]. The same study reports first visual signs at 3.7 mmol/L in a single patient, a value its own abstract restates as a general threshold indicating hemodialysis.

The qualification travels less well than the number. Formate clearance, not formate production, governs susceptibility. Elimination proceeds through the folate-dependent 10-formyl-tetrahydrofolate dehydrogenase pathway, whose capacity differs across species. Black and colleagues showed that hepatic tetrahydrofolate in the methanol-sensitive monkey is approximately 60% of that in the resistant rat, with correspondingly different formyl-tetrahydrofolate enzyme activities [37].

Johlin and colleagues extended the argument to human liver, demonstrating that the human pathway resembles the primate rather than the rodent configuration [38]. Rodents differ at two points rather than one: they oxidize methanol chiefly through catalase–peroxidase instead of alcohol dehydrogenase, and they clear formate through a folate-dependent pathway of substantially greater capacity than the primate one [37,38].

Both differences run in the same direction. Two consequences follow: rodent bioassays systematically understate human hazard, and folate status genuinely modifies individual susceptibility. The sources converge on the mechanism but diverge on its therapeutic exploitation—Zakharov and colleagues (2014) found no demonstrable protection against visual sequelae from folate administration in a large outbreak cohort [39], while accepting it as low-risk adjunctive therapy.

A second feature recurs, transposed, throughout this review. Reported elimination half-lives for the parent compound span roughly 2.5 to 87 h [4,33]. This is not measurement error: methanol elimination is saturable, approximating zero-order kinetics at toxicologically relevant concentrations, so the apparent half-life depends on the concentration at which it was measured and on whether ADH was pharmacologically blocked. A constant rate derived at one concentration cannot be transported to another. Section 7 shows that precisely this error—transferring a saturation-limited rate constantly across several orders of magnitude—underlies the environmental half-life figures the maritime literature has repeated for two decades.

### 4.2. Routes of Exposure: The Underweighted Dermal Pathway

The clinical literature is overwhelmingly one of ingestion because outbreak epidemiology is dominated by contaminated or counterfeit beverages [4,40]. Shipboard exposure has the opposite profile: ingestion is improbable, inhalation and skin contact routine. The evidentiary base is correspondingly inverted, leaving the dermal route the weakest-covered pathway for a fuel whose principal foreseeable human exposure is dermal. The controlled human data is nonetheless unambiguous. Batterman and Franzblau (1997) [41] exposed one hand each of twelve volunteers to neat methanol across sixty-five sessions and derived a mean absorption rate of 8.1 ± 3.7 mg cm^−2^ h^−1^. That figure agrees closely with an independent in vivo estimate of0.192 mg cm^−2^ min^−1^, equivalent to approximately 11.5 mg cm^−2^ h^−1^, reported by Dutkiewicz and colleagues in 1980 and reaching the present literature through the WHO Environmental Health Criteria monograph [35] and the acute exposure guideline documentation [42].

The primary record was not consulted directly. Both in vivo estimates substantially exceed in vitro diffusion-cell values—a divergence with regulatory consequence, since the permeation parameters embedded in quantitative risk models are usually in vitro-derived and will therefore underestimate real uptake. Two further findings are operationally decisive and rarely carried into maritime guidance. Blood methanol kept rising for roughly two hours after exposure ended and entered the circulation for about four, consistent with an epidermal reservoir: removing a worker from the source does not end the exposure. And the peak concentration from some twenty minutes of single-hand contact is comparable to that from a full shift inhaling 200 ppm, the threshold limit value [41]—a splash no gas detector will register can deliver a dose equivalent to an entire compliant working day.

The 2025 case reported by Alrougi and colleagues [5] supplies the maritime confirmation the earlier literature lacked: a 44-year-old seafarer performing a methanol wall-wash test in an enclosed space aboard a commercial vessel developed painless bilateral blindness within roughly twenty-four hours from combined inhalational and contact exposure, with poor visual outcome despite fomepizole and four hemodialysis sessions. Comparable occupational cases outside shipping [43] show the same pattern of delayed recognition. Against this, industry-facing documentation continues to foreground flammability: the stakeholder consultations underpinning current seafarer-training development recorded toxicity as the defining hazard for ammonia while framing methanol chiefly through flame invisibility, fire detection and PPE [44], and inspection guidance for methanol dual-fuel vessels, though it addresses exposure limits, is structured around fire and explosion control [1]. The asymmetry is documented rather than alleged and attributable to no single actor.

### 4.3. Ocular and Neurological Toxicity

Selective vulnerability of the retina, optic nerve and basal ganglia follows from the cytochrome oxidase mechanism. Optic nerve axons and their myelin sheaths carry few mitochondria and a limited cytochrome oxidase reserve, so a given degree of complex IV inhibition exhausts their oxidative margin first—the argument advanced by Sharpe and colleagues (1982) [45] and still accepted, retinal Müller cells are a second locus of formate action. Bilateral, symmetrical putamina necrosis, frequently hemorrhagic, is the characteristic central lesion, and the 2026 meta-analysis of cranial MRI findings pools its prevalence alongside optic nerve injury and mortality [46], while a restricted diffusion of the optic nerves on MRI has been described in case material following combined topical and inhalational exposure [47]. Long-term follow-up shows visual damage is frequently permanent and accompanied by retinal nerve fiber layer thinning [48,49].

Latency is a clinically dangerous feature. Onset typically falls between roughly ten and forty-eight hours after exposure, extended further when ethanol has been co-ingested, since ethanol competes for ADH and delays formate generation [4,32,33]. A worker may complete a shift, sleep, and wake blind—and aboard a vessel that window is frequently longer than the interval within which shore-based intervention could still alter the outcome.

One recent finding openly contradicts standard teaching. Methanol poisoning is taught as a high-anion-gap metabolic acidosis, yet the seafarer described by Alrougi and colleagues (2025) [5] presented in marked respiratory alkalosis at pH 7.607 with severe bilateral visual loss. Whether this reflects compensatory hyperventilation, exposure route or timing is unresolved, but a shipboard screening protocol keyed exclusively to acidosis would have missed this patient. Prognostic studies elsewhere similarly report that no single laboratory parameter performs reliably in isolation [50].

### 4.4. The Lethal Dose: An Unresolved Inconsistency

Values circulating for a lethal methanol dose differ by more than an order of magnitude, and the maritime literature—including our own earlier synthesis [2]—has propagated them without reconciliation. The clinical range is commonly given as 0.3–1 g/kg body weight, with the widely repeated statement that as little as 30 mL may cause permanent visual damage [33]. Standard poisoning references give a fatal oral dose of 30–240 mL, equivalently 20–150 g, with a minimum toxic dose near 100 mg/kg. Machiele’s (1987) [51] transport-fuel assessment, reproduced in Parris et al. (2024) [2], states 1–2 mL/kg. Moon’s (2017) [52] estimation from dose–response data reports 15.8–474 g per person, median 56.2 g, with 3.16–11.85 g per person sufficient to cause blindness.

These figures are not merely inconsistent; they answer different questions while being presented as answers to one—and that distinction is not new. The WHO/IPCS Environmental Health Criteria monograph stated in 1997 [35] that the human lethal dose is not known with certainty, that the figure of 0.3–1 g/kg is the minimum lethal dose in the absence of medical treatment, and that the minimum dose causing permanent visual defects is unknown. The monograph further identified concurrent ethanol ingestion, which delays entry into the metabolic pathway, and hepatic folate status, which governs the rate of formate detoxification, as the two principal determinants of individual susceptibility [35]. Moon (2017) [52] preserved the same separation quantitatively, reporting distinct ranges for the lethal and the blinding endpoint, and the clinical literature supplies the arithmetic caveat, converting explicitly at approximately 1 g to 1.2 mL. Four of the five grounds of divergence—endpoint, ethanol, folate status and treatment assumption—are therefore already documented in the primary literature; only the drift between per-person and per-kilogram reporting appears to be undocumented.

What has not happened is the application of that separation. Maritime risk documentation, our own earlier synthesis included [2], selects a single figure from this literature and propagates it into consequence modeling without recording which endpoint it represents or whether medical treatment was assumed. A more robust alternative is available and largely unused: the same 1997 monograph reports blood-concentration thresholds—with central nervous system effects above roughly 200 mg/L, ocular effects above 500 mg/L, and fatalities in untreated patients at 1500–2000 mg/L [35]—which are internally consistent because they bypass the absorbed-fraction, co-ingestion and unit-conversion problems altogether. For shipboard exposure scenarios, where any ingested volume would in any case be unknown, concentration-based endpoints are the sounder basis. It follows which of the circulating values should not be carried forward. The volumetric point estimates—the fatal oral dose of 30 to 240 mL, the 30 mL commonly cited for permanent visual damage, and the 1 to 2 mL/kg reproduced from the 1987 transport-fuel assessment—are single figures abstracted from untreated case series and are reported without an endpoint, without a treatment assumption, and without the co-ingested ethanol and hepatic folate status that the monograph identifies as the principal determinants of susceptibility. No body has formally withdrawn them, and they retain descriptive value, but on the criteria the 1997 monograph itself applied when it declined to certify a human lethal dose, they cannot serve as quantitative inputs to a consequence model, and they are reproduced above only to display the divergence. What survives that test is the endpoint-resolved reporting of Moon and the blood-concentration thresholds given here.

The same dissection resolves the second and more consequential contradiction. Clinical toxicology describes methanol as a substance of relatively low intrinsic toxicity [32,33]; the maritime gray literature describes it as an acute hazard to life. Both are correct within their own frame, and the frames differ in one variable: time to antidote. In a hospital with fomepizole and dialysis, operative toxicity is low. On a vessel days from definitive care, with no fomepizole in a standard medical chest and a latency that conceals the exposure until the therapeutic window has closed, it is high. The determinant is not intrinsic potency but the interval between exposure and ADH blockade. This reframing does not contradict the conclusion of Parris et al. [2] that methanol remains the milder option relative to heavy fuel oil and ammonia; it specifies the condition under which that mildness holds, and identifies antidote availability aboard methanol-fueled vessels as an actionable and currently unaddressed control.

### 4.5. Biomarkers of Exposure and the International Disharmony of Occupational Limits

Diagnosis rests on markers whose behavior over time is itself diagnostic. Serum and urinary formate are the direct indices of the toxic species. The osmolar gap is elevated early, when unmetabolized methanol dominates, and narrows as metabolism proceeds, while the anion gap widens reciprocally as formate accumulates. The corollary emphasized across recent reviews [4,33] is that a normal osmolar gap does not exclude poisoning in a late presentation—a pitfall of obvious relevance where transit to care is measured in days. The occupational case reported by Wu and colleagues illustrates the value of urinary sampling: urinary formate exceeded blood formate by more than an order of magnitude and remained detectable through successive dialysis sessions [43], arguing for it as a practical field biomarker where gas chromatography is unavailable. The published table required correction of its concentration units by a factor of ten [53]. The ratio reported here is unaffected, but the episode illustrates at the level of a single primary record the same loss of measurement conditions traced across compilations in Section 5.

Occupational limits, by contrast, are not harmonized, and Table 2 makes the spread explicit. The NIOSH recommended exposure limit [54,55] and the European indicative limit [56] converge on 200 ppm as an eight-hour value with a skin notation, and NIOSH sets a short-term limit of 250 ppm [54], which the American Conference of Governmental Industrial Hygienists also applies as a short-term value. The enforceable OSHA permissible exposure limit retains 200 ppm over eight hours but, following the remand of the 1989 air contaminants rule, carries neither the short-term limit nor the skin notation in binding form [57]. China’s GBZ 2.1 sets a time-weighted average of 25 mg m^−3^ and a short-term limit of 50 mg m^−3^ [58]—roughly an order of magnitude below the Euro-American consensus. The divergence is not a rounding artifact but a substantively different judgment about the acceptable margin for a skin-absorbed substance whose critical effect is irreversible. The convergence on the Euro-American side is, in turn, less independent than it appears: the OSHA limit was adopted from the 1968 ACGIH list [57], and the NIOSH and European values share that ancestry rather than deriving from separate evaluations. Four instruments state the same number; they do not constitute four assessments of it.

For shipping, the disharmony is structural rather than academic. A vessel may be flagged in one state, crewed by nationals of a second, managed from a third and bunkered in a fourth, so that no single occupational limit unambiguously governs the atmosphere of a methanol fuel-preparation room. European bunkering assessments accordingly fall back on the American IDLH value of 6000 ppm for emergency planning while applying European eight-hour limits for routine work [59,60], a hybrid that no single instrument authorizes. IMO MSC.1/Circ.1621 addresses toxic exposure through segregation, double barriers, detection and isolation rather than by specifying a numerical crew exposure limit [16]. The Sub-Committee on Carriage of Cargoes and Containers did not reach the substantive revision at its eleventh session in September 2025, but it re-established the intersessional working group and constituted a correspondence group to carry the work forward, giving the methyl and ethyl alcohol guidelines explicit priority over the other low-flashpoint fuels [61,62,63]. The stated purpose of that revision is to secure consistency in the approach to toxicity requirements [64]. This section therefore closes on an open regulatory question.

The unifying finding is that human methanol toxicity is not methanol toxicity at all but formate toxicity, gated by a folate-dependent clearance pathway whose capacity differs between organisms. That formulation is deliberately general, and Section 7 exploits it: the same bottleneck explains the distribution of sensitivity across aquatic taxa, where organisms differ in their capacity to oxidize formate much as monkey and rat do [6]. The apparent contradiction between a compound that blinds humans and one that aquatic testing classifies as practically harmless is not a contradiction between two toxicologies, but a single toxicology observed in organisms with different formate-handling capacities, at concentrations differing by many orders of magnitude.

## 5. Environmental Fate and Behavior of Methanol in the Marine Environment

### 5.1. Dispersion Physicochemistry and the Inapplicability of Oil-Spill Models

Three properties govern methanol’s behavior after release, and on these the literature is unanimous: complete miscibility with water, a specific gravity near 0.792, and a vapor pressure of roughly 126 mm Hg at 25 °C [11], equivalent in the arithmetic of this review to some 168 hPa and consistent with the 127.9 hPa reported at 20 °C in Section 3.1. ITOPF places methanol among the substances of the Standard European Behavior Classification that dissolve while also evaporating, so a release floats and spreads only briefly before partitioning into the water column and the atmosphere rather than persisting as a coherent slick [8,65]. Kass and co-workers reach the same conclusion from property-based prediction [13], and the later NOAA and P&I syntheses reproduce it [12,66].

The operational consequence is that the standard oil-spill toolkit has no object on which to act: booming, skimming and shoreline cleanup all presuppose a floating phase that methanol does not maintain, which Le Masurier frames as a change of response paradigm, not an adaptation of technique [65].

Sources diverge, however, on the quantity that replaces slick area as the object of prediction. The figure recurring across the maritime literature is dilution below one percent, described as a non-toxic level [8,12,66]. Its provenance is traceable. Machiele’s (1989) [67] hypothetical simulations, reported in the 1999 assessment for the American Methanol Institute, yielded 0.36 percent within an hour of a 10,000-tonne open-sea release [11]. Two qualifications visible in the primary source disappear downstream. First, one percent corresponds to roughly 10,000 mg L^−1^, a value anchored in acute lethality data for fish, not in any no-effect threshold. Second, both presuppose open-water mixing energy, not a harbor basin or a stratified fjord. The 1999 assessment cautioned that its tabulated fate parameters were order-of-magnitude estimates of uncertain field provenance, to be applied only with care [11]—a caution absent from every maritime source that carries the figure forward.

### 5.2. Volatilization and the Henry’s Law Constant

The sharpest contradiction in the corpus concerns volatilization. ITOPF states that volatilization “plays a major role in the decomposition of methanol in the marine environment” [8]. The P&I literature carries the sentence almost unaltered [66], which is not independent corroboration: that article acknowledges ITOPF’s contribution to its research and reproduces the same property table, the same behavior classification and the same one-to-seven-day half-life.

The physicochemical literature states the opposite for dissolved methanol. The Henry’s law constant of methanol is approximately 4.55 × 10^−6^ atm m^3^ mol^−1^ [11], equivalent to 0.461 Pa m^3^ mol^−1^—the value adopted at 25 °C in the OECD SIDS initial assessment [9]—and consistent with the compilation of Sander(2023) [27]. Division by RT at 298.15 K gives a dimensionless air–water partition ratio of 1.86 × 10^−4^. The consequence is stated by the assessing body rather than inferred here: OECD concludes that volatilization is not a significant removal process for methanol from the aquatic compartment [9]. The same constant is put to a second and opposite use within that same document, where its low value is invoked to justify accepting acute LC50 values obtained from unsealed test vessels on the ground that nominal and measured concentrations may be equated [9]. The consequences of that second use are taken up in Section 7. Modeled volatilization half-lives for dissolved methanol under lake conditions run to tens of days [11].

The two positions are reconcilable, and the reconciliation is already present in the primary sources rather than supplied here. Evaporation from a floating pool, before dissolution is complete, is rapid, and it is what Kass and colleagues model for a surface release [13], volatilization of methanol already dissolved in the water column is slow, and it is what the Henry’s law constant governs [11,27]. The error in the ITOPF formulation is therefore not one of imprecision. Two things are wrong with the sentence. Volatilization is a phase transfer, not a decomposition: the molecule that leaves the water column is chemically unchanged. And volatilization, unlike evaporation from a floating pool, is the specific process the Henry’s law constant governs—the process the assessing body concludes is not significant for this compartment.

The salting-out effect of seawater raises Henry’s law constants for short-chain alcohols marginally; the only experimentally determined marine salting-out constants located for this review were obtained for ethanol [68], and their application to methanol is stated here as an assumption, not a measurement. The effect is in any case too small to alter the conclusion. Once methanol has entered the water column, atmospheric loss is not an escape route for the dissolved mass.

### 5.3. Biodegradation: Provenance and Limits of the One-to-Seven-Day Half-Life

Biodegradation is presented throughout the maritime literature as the dominant loss process, with a surface-water half-life of one to seven days [8,12,65,66]. The same characterization has migrated into peer-reviewed assessments of methanol as a marine fuel [30]. The reproduction chain for the figure can be reconstructed in full and is shown in outline in Figure 1. Its upstream source is the compilation of degradation rate constants by Howard et al. (1991) [10], in which the surface-water entry is expressly derived from unacclimated aqueous aerobic biodegradation assays. The 1999 assessment for the American Methanol Institute tabulates the value and identifies that compilation as its origin [11]. ITOPF cites Howard directly [8,10,65], NOAA and the P&I literature cite ITOPF [8,12,66]. At each transfer, the assay qualifier is dropped, and by the final link the range is presented as a property of methanol in seawater. The transfer itself is disputed at the source: GESAMP states that biodegradation proceeds less rapidly in marine than in freshwater systems and accordingly prefers marine test designs [28,69].

A numerically similar range arises independently in marine biogeochemistry, which makes the conflation easy to overlook. Dixon and colleagues measured microbial methanol oxidation in northeast Atlantic surface seawater at 2.1–8.4 nmol L^−1^ d^−1^, with a maximum velocity of up to 29 nmol L^−1^ d^−1^ and a half-saturation constant of 9.3 nM in productive coastal water [7], and reported assimilation rates in Atlantic upwelling systems around an order of magnitude above those of oligotrophic gyres [70]. Turnover times computed from these rates span roughly one to seven days. Crucially, these are ambient-concentration measurements: in situ methanol is nanomolar, comparable to the half-saturation constant, so uptake is approximately first-order and a turnover time is meaningful.

That condition fails entirely at spill concentrations. A release producing grams per liter corresponds to tens of millimolar to molar concentrations, six to eight orders of magnitude above the half-saturation constant, where Michaelis–Menten kinetics become zero-order and removal proceeds at the maximum velocity. At a measured maximum velocity of 29 nmol L^−1^ d^−1^, gram-per-liter concentrations cannot be removed on any operationally relevant timescale. The rate that supports a one-day turnover at nanomolar concentration therefore does not scale, and neither the biogeochemical nor the maritime literature has connected the two regimes.

The point of failure can be stated as a number of orders of magnitude, because the Michaelis–Menten form fixes it. First-order behavior requires a substrate concentration well below the half-saturation constant, and a first-order rate underestimates the removal time by a factor of one plus the ratio of the two. At a tenth of the half-saturation constant, about 0.9 nM, the error is ten percent and the reported turnover time holds. At the constant itself, 9.3 nM, the true removal time is already double the first-order prediction; one order of magnitude above it, near 3 µg L^−1^, the prediction is out by a factor of eleven, and three orders above it, near 0.3 mg L^−1^, by a factor of a thousand. The parameter has therefore lost its meaning roughly one order of magnitude above the half-saturation constant and is wrong by three orders at three—all of this still three to four orders of magnitude below the gram-per-liter concentrations a release produces. At 1 g L^−1^, which is 31 mmol L^−1^ and some 3.4 million times the half-saturation constant, the kinetics are zero-order and removal proceeds at the measured maximum velocity of 29 nmol L^−1^ d^−1^ irrespective of concentration, so clearance of that concentration would occupy of the order of 10^6^ days against the one to seven quoted. That last figure is not offered as a prediction of persistence: microbial populations grow on an enriched substrate, so the measured maximum velocity is a property of the biomass present when it was measured rather than a ceiling, while the inhibition described below acts in the opposite direction. What the arithmetic does establish is that, between the regime in which the one-to-seven-day figure was measured and the regime in which it is quoted, lie six to eight orders of magnitude in concentration and at least five in predicted clearance time, so the measured parameter carries no information about the spill case.

A second constraint is inhibition. Alcohols above approximately 100,000 mg L^−1^ are generally toxic to microbial populations; exposure to 19 percent methanol reduced an aquifer microbial population by four orders of magnitude [71], and hydrocarbon biodegradation was impaired at 14,000 mg L^−1^ methanol, both as reported in the 1999 assessment [11]. The primary records [71,72] have not been consulted directly and are cited here at second hand. The second of those attributions does not survive checking. The study to which the 1999 assessment credits the inhibition finding examined methanol biodegradation in soil microcosms at concentrations up to 1000 mg/L and concluded that groundwater contamination problems should be minimal given the compound’s susceptibility to biodegradation. It reports neither an experiment at 14,000 mg/L nor any effect on hydrocarbon biodegradation [72]. The same assessment cites that study accurately in its own section on subsurface biodegradation [11].

The transmission failure in this chain is therefore not confined to the loss of test conditions: at one link the identity of the source is misassigned, and the finding attributed runs opposite to the one published. The same pattern recurs in Section 8.2 at the level of the regulatory literature. Katsumata and Kastenberg (1996) [73] report the same inhibition logic for subsurface releases of methanol fuel: near-source concentrations may suppress the very organisms invoked to remove them. The system is not the marine one, and no equivalent determination exists for seawater. Biodegradation is thus slowest precisely where the released mass is greatest, which inverts the assumption embedded in the half-life. This is the same saturation logic identified in Section 4 for clinical elimination, where the reported half-life ranges from 2.5 to 87 h as a function of dose; it recurs here as a structural feature of the evidence base rather than as coincidence.

The 1999 assessment closed by recommending that recorded methanol releases be analyzed to confirm that the substance behaves as predicted, precisely because high near-source concentrations might inhibit degradation [11]. That recommendation remains unfulfilled: no post-incident field verification appears in the literature reviewed here.

### 5.4. Oxygen Consumption and Hypoxia

The complete oxidation of methanol consumes 1.5 mol of oxygen per mole of substrate, giving a theoretical oxygen demand of 1.50 kg O_2_ per kg of methanol. Fully saturated temperate seawater holds roughly 8 mg L^−1^ of dissolved oxygen, so one kilogram of methanol is stoichiometrically capable of exhausting the oxygen contained in about 190 m^3^ of seawater, and a bunker-scale release of 100 t that of some 1.9 × 10^7^ m^3^, before any allowance for reaeration or mixing.

This oxygen demand is not a novel inference. The 1999 assessment predicted that a large release into an enclosed area would deplete the oxygen required to sustain aquatic life [11], and both ITOPF and NOAA acknowledge hypoxia in sheltered waters as the principal environmental mechanism while expecting it to be short-lived [8,12]. Experimental support is direct: amendment of coastal seawater with methanol produced an approximately twofold increase in oxygen consumption alongside elevated bacterial growth [74].

The empirical analog is stronger still for ethanol, a chemically and metabolically comparable fuel alcohol. NOAA reports that ethanol is practically non-toxic to most aquatic species over 24-h exposures and that fish kills following spills in fresh water are attributable to oxygen depletion, not to direct toxicity, with hypoxic plumes propagating tens of kilometers downstream over several days [75,76]. Hypoxia is conventionally defined at dissolved oxygen below 2 mg L^−1^ [77]. The ethanol case demonstrates the mechanism, magnitude and spatial extent that the methanol literature still treats as speculative.

What is absent is quantification. No published simulation couples a methanol release scenario, concentration-dependent degradation kinetics and dissolved-oxygen dynamics for a port basin, a fjord or a stratified coastal water body. The expectation that hypoxia will be short-lived [8] is asserted rather than modeled, and it rests on the same degradation rates whose extrapolation Section 5.3 shows to be unsupported. The ecotoxicological consequences of this pathway are developed in Section 7. The phase-dependent fate of released methanol and the associated oxygen-demand pathway are summarized in Figure 3.

### 5.5. Hazard Classification Against the Actual Risk Profile

Methanol is regulated in bulk carriage as methyl alcohol under chapter 17 of the IBC Code, where it carries pollution category Y and ship type 3 [78,79]. Category Y denotes substances presenting a hazard sufficient to justify limitation on the quality and quantity of operational discharge [78]. The pollution category derives from the GESAMP hazard profile, whose evaluated columns cover bioaccumulation, biodegradation, acute and chronic aquatic toxicity, and human health endpoints [28,69].

The 2019 amendments to the Code, in force since January 2021, show the classification machinery working. Methanol’s hazard column moved from pollution-only to combined safety and pollution; vapor detection was extended from flammable to flammable and toxic; gauging was tightened from restricted to closed; and the toxic-product provisions of Section 15.12 of the IBC Code became applicable [79,80]. Every one of those revisions addresses human toxicity. The pollution category and the ship type were left unchanged. The framework therefore does revise methanol’s classification when new data arrives but only along the safety axis.

Oxygen demand appears in none of the thirteen endpoints. E2, the only column addressing behavior in the marine environment, is evaluated on solubility, melting point, vapor pressure, specific gravity and viscosity—the physics of slicks and seabed blanketing—while biodegradation enters as the binary flag A2, scored on oxygen depletion reaching sixty percent of the theoretical maximum [28,69]. The same oxygen consumption thus counts as evidence that a substance is benign, never carried forward as a hazard. Category Y reflects low acute toxicity but is silent on the pathway the same literature calls principal. Methanol used as a fuel falls outside Annex II altogether, governed by the non-mandatory guidelines of MSC.1/Circ.1621 [16], which address containment and crew exposure and set no marine environmental fate criterion. Lee and co-workers report a structurally identical mismatch on the safety side and call for environmental impact assessment criteria covering marine leakage of alternative fuels [3], the codification program of the IMO carriage sub-committee provides the vehicle [61,62,63].

### 5.6. Synthesis and Research Gaps: Environmental Fate

On the endpoints for which measured values exist, methanol remains environmentally preferable to heavy fuel oil: it leaves no persistent residue and no oiled shoreline, bioconcentration factors are below 10, and ready biodegradability reaches 76 to 82 percent within five days [9]. The comparison with ammonia is asserted more often than it is measured; no source located for this review reports acute aquatic toxicity, persistence and bioaccumulation for both fuels on a common basis, and the ranking is carried here no further than the evidence supports. What evidence does not support is the confidence with which its fate parameters are stated. Five gaps follow directly.

First, no measurement of methanol biodegradation kinetics in seawater exists at spill-relevant concentrations; the available half-saturation and maximum-velocity constants describe nanomolar biogeochemistry [7,70]. Second, no coupled hydrodynamic, biokinetic and dissolved-oxygen model has been published for confined or stratified receiving waters. Third, no post-incident field verification exists, twenty-seven years after it was formally recommended [11]. Fourth, the regulatory framework applying to methanol as a fuel contains no environmental fate criterion at all [3]. Fifth, the modeling tool in routine use cannot represent the pathway. A 2026 consequence analysis of four alternative fuels reports that the dispersion software applied throughout maritime risk assessment does not account for solubility, assumes the entire released mass is available for atmospheric dispersion, and cannot simulate liquid leaks in piping at all; the authors record this as a limitation specific to methanol and proceed to rank it the safest transitional fuel [81].

The aquatic compartment is not assessed and found benign. It is not assessed. The critique here targets the evidentiary basis of the sector’s fate claims, not the comparative ranking those claims support.

## 6. Exposure Scenarios and Pathways: From Bunkering to Accidental Release

### 6.1. A Taxonomy of Exposure Situations

Section 4 and Section 5 established what methanol does to the human organism and what becomes of it in seawater. The intervening question is operational: under which circumstances does contact occur, and who is standing where when it does? The taxonomy adopted here is not constructed for this review. Ismail, Metwalli and Alamoush (2025) [17] developed and published a hazard-based coding system that separates toxicity into four discrete exposure codes—dermal (T1), ocular (T2), inhalation (T3) and ingestion (T4)—alongside flammability and explosivity codes, and applied it to thirty investigated methanol accidents. That published scheme is used as the organizing frame, and its results are reported as its authors report them.

Across the fifty-three coded hazard events, physical hazards accounted for roughly four-fifths of occurrences and the health-hazard routes for the remaining fifth; tank explosion alone represented some 38 percent of coded events and was implicated in fifteen of the nineteen recorded fatalities [17]. The distribution inverts, however, when severity is conditioned on occurrence: inhalation appeared in only about 7.5 percent of coded sub-hazards yet carried a case-fatality proportion of roughly one-half within the incidents in which it featured, while dermal exposure appeared once and produced only mild injury [17]. The same authors state the principal limitation themselves—twenty-four of the thirty reports originate from land-based industrial files and only three from the IMO shipping database, so the distribution is extrapolated to the maritime setting, not derived from it.

Mapping personnel onto that frame gives five recurring situations. Normal operation exposes engine-room personnel to low-level vapor and, on pipework failure, to a rapidly developing cloud in a confined volume. Bunkering and simultaneous operations place deck crew, bunker-vessel personnel and terminal operators alongside pressurized flanges and hoses. Maintenance and tank entry combine liquid contact with a vapor-laden atmosphere. Collision or grounding produces uncontrolled release with no preparatory control. Incident response introduces the responders, whose exposure is deliberate rather than accidental.

### 6.2. Bunkering as the Dominant Recurring Scenario

Bunkering matters disproportionately because it recurs. The volumetric energy density of methanol, at approximately 15.8 GJ m^−3^, is well under half that of marine gas oil, so an equivalent range demands roughly two to two and a half times the tank volume, and where tank volume is constrained the deficit is met by more frequent transfers [2,3]. Every transfer is a full cycle of connection, transfer, draining, purging and inerting, and each step is a discrete opportunity for release. The regulatory scaffolding has consolidated rapidly: ISO 6583:2024 fixed fuel quality at custody transfer while explicitly devolving health and safety practices to the user [26], class and industry guidance place a mandatory risk assessment at the center of bunkering approval [82,83] and a European standard now covers inland-waterway transfer [84].

Quantified risk modeling has advanced in parallel, and the sources converge on method while diverging on endpoint. Khan, Zhang and Pan (2026) [85] constructed a fault-tree to system-dynamics to Bayesian-network chain for a methanol bunkering station in Shanghai, ranked hazard factors by mutual information, and modeled consequences with a fire-and-thermal code, stating that the coupling of bow-tie analysis, dynamic simulation and Bayesian networks for temporal methanol bunkering risk had not previously been reported. Their individual and societal risk figures are, however, derived from thermal effects. The comparable LNG work of Kong et al. (2023) [86] likewise quantifies the combustible vapor cloud envelope. Neither is deficient on its own terms; the point is that the apparatus applied to methanol bunkering has so far been calibrated against ignition, not inhaled dose.

### 6.3. Denser-than-Air Vapor: Which Sources Quantify and Which Only Describe

Methanol vapor has a relative density of 1.11 against air [87]. The qualitative consequences are stated consistently across the guidance literature—a cloud that does not rise, travel along decks into low-lying and confined volumes, and flashback from a remote ignition source—which also notes that the flame is difficult to see in daylight [17,87,88]. Quantification is far thinner and almost entirely recent. Sun et al. (2025) [19] simulated liquid methanol release in the main engine compartment of a 249.8 m vessel over a 35 by 25 by 4 m domain and reported that the cloud hugs the floor precisely because the vapor is denser than air; that a 12 cm orifice fills the compartment within fifteen seconds and drives peak vapor height to 2.95 m, some 86 percent above the six-centimeter case; and that raising ambient temperature from 298 to 328 K extends diffusion distance by about 6.7 percent.

The comparison that a review must draw is what those outputs are referred to as against. Sun and colleagues report cloud geometry—extent, height, accumulation against bulkheads—and cite an acute-poisoning range of 39.3 to 65.5 g m^−3^ in their introduction, but that toxicological figure is never mapped onto the simulated concentration field, and no breathing-height time-concentration profile is presented [19]. The equivalent exercise has been performed for ammonia. Liu and co-workers [89] simulated ship-to-ship bunkering leaks against toxic thresholds, reported lethal zones extending tens of meters across the deck for vertical releases in crosswind, demonstrated that Gaussian plume models are inadequate within the near field because of hull and superstructure wakes, and recommended vessel-specific computational fluid dynamics before any bunkering operation is certified as safe. A 2026 parametric study established safety zones for LNG, ammonia and hydrogen under standardized conditions [90], methanol is not among the fuels compared. The methodology for toxicant-referenced zoning therefore exists and has been exercised on alternative fuel whose acute toxicity is more conspicuous, but not on methanol.

The practical residue is that safety-zone criteria remain anchored to flammability. Port bunkering regulations define the zone as the area within which ignition sources are controlled and access is restricted, sized from vapor dispersion calculated for the largest credible leak, with a vertical extent and a minimum security radius of twenty-five meters [91].

Two independent sources make parallel observations about the IMO interim guidelines. Ismail et al. (2025) [17] record that no threshold limit value or toxicity alarm is stipulated and that lethal vapor concentrations can therefore accumulate well before flammable limits are approached. Lee and co-workers [3] state the same structurally, noting that the quantitative triggers for automatic emergency shutdown remain twenty percent of the lower explosive limit for alarm and forty percent for shutdown even though the guidelines explicitly recognize the toxicity hazard, which they characterize as a disconnection between the dominant assumed risk and the actual triggering logic of the safety system.

The published numbers bear this out without further interpretation: the lower explosive limit is reported to be between 55,000 and 60,000 ppm, so a twenty-percent-LEL alarm point falls between roughly 11,000 and 12,000 ppm. The spread reflects the flammable range itself, given as 6 to 36.5 vol % in the comparative appendix [3] and slightly lower elsewhere; the conclusion is unaffected, since the alarm point exceeds the immediately dangerous concentration at either bound. Whereas the immediately dangerous concentration is 6000 ppm and the eight-hour AEGL-3 is 1600 ppm [42,87]. The relationship between toxicological reference concentrations and the current flammability-based methanol detection thresholds is illustrated in Figure 4.

### 6.4. The Combined Pathway: Dermal and Inhalation Exposure Acting Together

Section 4 established that cutaneous uptake alone, at roughly 8.1 mg cm^−2^ h^−1^ in vivo, can deliver over some twenty minutes on one hand a blood concentration comparable to a full shift at 200 ppm [41]. Deck and tank conditions place liquid contact and vapor inhalation in the same place at the same moment, so the two routes are not alternatives but co-occurring contributions to one internal burden.

That co-occurrence is documented clinically. Alrougi et al. (2025) [5] reported a forty-four-year-old seaman who conducted a methanol wall-wash test in an enclosed space aboard a chemical carrier. The exposure is recorded as dermal contact to arms and face together with brief inhalation and was followed by bilateral loss of vision that persisted through fomepizole administration and four hemodialysis sessions, with an atypical respiratory alkalosis instead of the expected metabolic acidosis. The authors state that this combined route, while less frequent than ingestion, is increasingly recognized in occupational settings, citing the inhalational series of Shen et al. (2023) [92]. Mojica et al. (2020) [47] had earlier described vision loss following chronic topical and inhalational exposure, and Givens et al. (2008) [93] compared exposure routes as reported to poison control centers.

What is absent is quantitative aggregation. No source located here sums the dermal and inhalational contributions into a single internal dose or body burden for a defined shipboard scenario. The coding system of Ismail et al. (2025) [17] treats dermal and inhalation as separate codes with separate frequencies, which is appropriate for incident classification but forecloses summation. Occupational biomonitoring guidance notes the converse difficulty: a biomarker aggregates every pathway simultaneously and cannot by itself apportion the internal dose among routes [94]. Neither observation substitutes for the other, which is why a route-resolved aggregate model would be required and why no exposure figure presently feeding maritime risk assessment can be assumed to represent the whole. This is stated here as a research gap, not as an analysis performed within this review.

The modeling frameworks that such an aggregate would require already exist outside the maritime literature, and naming them is more useful than restating the absence; they are identified here as starting points, not applied. The dermal contribution must first be converted from a flux into an absorbed mass, and the quantitative structure–activity model of ten Berge, implemented as the IH SkinPerm tool, does exactly that, estimating a maximum steady-state absorption in mg cm^−2^ h^−1^ and a lag time from molecular weight, octanol–water partition coefficient and water solubility [95]; this is the transformation the 8.1 mg cm^−2^ h^−1^ determination of Section 4.2 requires before it can be added to anything. The two contributions must then be summed within a structure that accepts more than one route, and the ECETOC Targeted Risk Assessment tool is the established occupational instance, estimating worker inhalation and dermal exposure in a single framework and expressing both against a common internal reference value; its dermal component has been validated against measured exposure levels and shown to be biased toward overestimation at the low end, a property a shipboard application would have to carry explicitly rather than silently [96]. The summed external dose must finally be translated into an internal one, and for methanol, this requires no new model: the multicompartment toxicokinetic model of Bouchard and colleagues already represents the disposition of methanol and formate in humans under differing exposure scenarios and yields blood and urinary predictions against which a route-resolved shipboard estimate could be tested [97]. The aggregate exposure pathway framework supplies the organizing logic for assembling the three into a single target-site estimate and for recording which links rest on data and which on assumption [98]. What is missing is therefore not a method but its parameterization: none of these has been populated with the contact areas, task durations and vapor concentrations of a bunkering transfer, a wall-wash test or an enclosed-space entry, and none of those quantities is beyond routine shipboard measurement.

### 6.5. Responders, Bystanders and Shore Personnel

The exposed population extends beyond the certificated crew, and the instruments governing it diverge sharply. Numerical thresholds for non-occupational populations exist and are published: interim acute exposure guideline levels at eight hours of 270, 520 and 1600 ppm for the three tiers, sixty-minute emergency response planning guidelines of 200, 1000 and 5000 ppm, and an immediately dangerous concentration of 6000 ppm [42,87,99]. The distinction between the two regulatory frames is drawn by the issuing body itself: occupational limits are written to protect healthy adult workers across a working lifetime, whereas the response guidelines are written to protect the general public from rare, unanticipated, short-term releases [99].

Odor provides no reliable margin. The NIOSH emergency response card places distinct odor awareness at 8.9 ppm and detection across roughly 100 to 1500 ppm [17,87], a range that straddles the 200 ppm occupational limit itself; other compilations report thresholds spanning a wider interval still, and Section 8.4 takes up the consequence. The response doctrine is correspondingly conservative, requiring fully encapsulating protection above the second-tier guideline or the immediately dangerous concentration [87].

None of these thresholds are invoked by the maritime instruments that govern methanol bunkering. Ismail and colleagues [17] document the corresponding medical gap directly: neither the international medical guide for ships nor the national equivalent addresses methanol toxicity other than by ingestion, leaving inhalational and ocular exposure without shipboard first-response guidance. Dock workers, passengers on weather decks and small-craft traffic within the port basin consequently fall between the two frames: the occupational limit is written for the crew, and the general-population family is referenced by no maritime instrument reviewed here.

### 6.6. Synthesis and Research Gaps: Exposure Scenarios

The sources converge on three propositions: that methanol vapor is denser than air and accumulates in confined and low-lying spaces; that bunkering is the highest-frequency contact scenario; and that the accident record is dominated by fire and explosion, not poisoning [17,19,85,100,101]. They diverge, or more precisely fail to engage, on which endpoint should govern design. Explosion produced most recorded fatalities and attracts most modeling effort, yet inhalation, when it occurred, proved lethal in half the incidents in which it appeared [17].

Four gaps follow, each either stated by a published source or evident from its absence. First, no computational or quantitative risk study located here reports methanol concentrations at breathing height against occupational or general-population thresholds, although precisely this has been done for ammonia [19,89,90]. Second, no aggregate dermal-plus-inhalation dose model exists for shipboard scenarios, despite clinically documented co-occurrence [5,47]. Third, safety-zone criteria remain flammability-anchored while toxicological thresholds lie roughly an order of magnitude below the flammable ones [42,87,91]. Fourth, the call by Lee and colleagues [3] for environmental impact assessment criteria for marine leakage of alternative fuels has a human-exposure analog that is equally unmet.

The scenarios described here are the input to the environmental analysis. What is not lost to the atmosphere or to combustion enters the water column as the source term for the fate pathways examined in Section 5 and ultimately determines the concentrations to which the organisms considered in Section 7 are exposed.

## 7. Ecotoxicological Effects on Marine and Aquatic Organisms

### 7.1. Acute Toxicity Across Trophic Levels

Methanol is consistently classified as a substance of low acute aquatic toxicity, a characterization that rests on a comparatively narrow and chronologically remote experimental base. Reported median lethal concentrations span three orders of magnitude across taxa. For teleost fish, 96-h LC50 values fall between 15,400 and 29,400 mg/L; the blue mussel *Mytilus edulis* responds at a comparable level of 15,900 mg/L; and for the green alga Chlorella pyrenoidosa, the intergovernmental assessment reports a calculated growth-inhibition EC50 of 28,440 mg/L over 10 to 14 days [9], with the primary determination [102] not having been retrievable during preparation. Within freshwater assemblages, the cladoceran *Moina micrura* emerges as markedly more sensitive, with a 96 h LC50 of 4.82 g/L, followed by *Oreochromis mossambicus* at 15.32 g/L and the oligochaete *Branchiura sowerbyi* at 54.89 g/L [6].

The primary determination just flagged is the 1996 acute test on Mytilus edulis, and its standing warrants emphasis rather than a parenthesis. It is the only acute marine value in the corpus; its primary record could not be retrieved during preparation; and it reaches this review through a secondary account published in 2004. It is accordingly the weakest evidence relied on anywhere in this review, and every statement made here about acute toxicity in the marine compartment inherits that weakness. It should not be quoted as an independently verified marine endpoint, and no risk assessment should treat it as one. The remaining acute values are freshwater, so the marine compartment of a fuel now entering service is represented in the accessible literature by one determination made thirty years ago whose primary record no present reader can inspect.

The dispersion of these values is not random noise. Kaviraj and colleagues advanced a mechanistic explanation that has been largely overlooked in the maritime literature: acute sensitivity to methanol tracks the capacity of the organism to metabolize formate, with the least tolerant taxa being those least able to process it [6,38]. This proposition reframes the entire question. Methanol toxicity in aquatic systems is not a property of the compound in isolation but of the enzymatic architecture of the receptor organism—precisely the same principle that governs its exceptional toxicity in humans, where the absence of an efficient folate-dependent formate clearance pathway distinguishes primates from rodents. Under this reading, the frequently drawn contrast between “highly toxic to humans, benign to marine life” is not a contradiction between two substances but a single toxicological mechanism expressed across organisms of differing metabolic competence.

The comparative literature nonetheless diverges sharply on how to communicate this. Technical assessments prepared for European policy audiences state that methanol is not toxic to aquatic organisms and produces no long-lasting effects [103]. Marine insurance and response bodies adopt a more guarded position, holding that methanol remains capable of harming fish, crustaceans and microorganisms despite its biodegradability and solubility [66,104]. A third position, articulated in port-level environmental communications, singles out marine mammals as susceptible to acute exposure while treating other aquatic organisms as comparatively less sensitive [105]. These are not reconcilable as stated. The present review interprets the divergence as an artifact of reporting the same small dataset against different reference thresholds, compounded by the absence of any taxon-specific evidence for marine mammals, for which no controlled exposure data exists.

### 7.2. Sublethal and Chronic Endpoints

Acute lethality is a poor proxy for ecological consequence, and this is where the evidence base is thinnest. Feeding-rate bioassays demonstrate a significant suppression of appetite in fish at 736.10 mg/L and above, with parallel effects on growth and reproduction [6]. The same program derived a no-observed-effect concentration of 23.75 mg/L for the most sensitive freshwater endpoints [6]—a value roughly 650-fold below the fish LC50 and one that, notably, does not appear in any of the maritime technical reports reviewed here. Behavioral responses in bivalves—sluggish movement, extended siphon, delayed reflexes—were recorded during acute exposures but never quantified; the observation reaches this review only through the secondary account of Kaviraj et al. (2004) [6], the primary report [102] not having been retrievable during preparation.

Three consequences follow. First, sublethal thresholds are approximately three orders of magnitude lower than the lethal values on which regulatory reassurance is built. Second, essentially all these data derive from freshwater species; no equivalent marine chronic dataset exists. Third, no predicted no-effect concentration (PNEC) for seawater has been formally derived and published for methanol under a recognized species-sensitivity-distribution framework. Statements that a spill would or would not exceed an “environmentally safe concentration” [66] therefore rest on a threshold whose derivation is not traceable in the peer-reviewed record.

### 7.3. The Indirect Pathway: Oxygen Demand and Localized Hypoxia

The prevailing view across recent technical assessments is that direct toxicity is not the principal ecological hazard. The rapid microbial degradation of a methanol release can drive sheltered waters into hypoxic or anoxic conditions, an effect characterized as short-lived given the compound’s non-persistence [8,66]. The mechanism is well established for the structurally analogous ethanol, where releases to fresh water produce fish kills through elevated biochemical oxygen demand rather than through direct toxicity, with hypoxic plumes capable of traveling downstream and remaining lethal for days [75]. The transfer to a marine setting is by analogy, not by measurement. Complete oxidation of one kilogram of methanol consumes approximately 1.5 kg of dissolved oxygen; against typical seawater saturation of 8 mg/L, one kilogram is stoichiometrically sufficient to exhaust the oxygen in roughly 190 m^3^ of saturated seawater, so the volume affected by a bunker-scale release is set by the exchange rate of the receiving basin rather than by the released mass alone.

The evidence for this pathway is nevertheless entirely inferential. No published simulation couples a methanol release to a validated dissolved-oxygen model, although the modeling infrastructure exists and has been applied to nutrient-driven estuarine hypoxia [106]. This is a tractable and high-value research gap: adapting an established eutrophication module to an acute organic-carbon pulse would replace assertion with quantification and would be directly relevant to enclosed and semi-enclosed bunkering locations.

### 7.4. Microbial Degradation Kinetics: A Scale Mismatch

The claim that dominates every recent maritime source—an aqueous half-life of one to seven days, attributed principally to biodegradation [8,12,66]—warrants a closer examination against the marine microbiological literature, which the maritime assessments do not cite.

Methanol is a natural constituent of seawater and a substrate for a well-characterized methylotrophic community. Bacterial methylotrophy proceeds through periplasmic methanol dehydrogenase, encoded by xoxF where lanthanide-dependent and mxaF where calcium-dependent, oxidizing methanol to formaldehyde before further oxidation or assimilation via the serine or ribulose monophosphate cycles [107,108]. Rate measurements are available. Biological oxidation in northeast Atlantic surface waters proceeds at 2.1 to 8.4 nmol per liter per day, with a maximum velocity of up to 29 nmol per liter per day and a high-affinity half-saturation constant of 9.3 nM in productive coastal waters [7]. In coastal upwelling systems, as much as 57% of methanol taken up is assimilated into cellular carbon, against roughly 12% in shelf waters and about 1% in oligotrophic gyres [70].

These parameters describe a system operating at nanomolar concentrations. A methanol release generating water-column concentrations in the gram-per-liter range exceeds the reported half-saturation constant by six to eight orders of magnitude. Under Michaelis–Menten behavior, the community is saturated, degradation proceeds at a fixed maximum rate independent of concentration, and the effective clearance time scales linearly with the mass released rather than following first-order decay. A half-life derived from ambient turnover cannot legitimately be transferred to spill conditions. Two further considerations reinforce this.

Formaldehyde, the obligate first intermediate, is substantially more toxic than the parent compound and is a recognized inhibitor of the very organisms producing it, raising the prospect of autoinhibition at high substrate loading. And where oxygen is exhausted, anaerobic pathways take over: in sulfate-rich Mediterranean sediments methanol has been identified as the dominant methanogenic substrate, accounting for up to 98% of methane produced in surface sediment [109]. Whether an oxygen deficit following a release would redirect the remaining methanol along that pathway has not been examined.

The consensus figure is therefore not wrong so much as misapplied—derived from steady-state biogeochemistry and imported into an accident scenario without adjustment. This, rather than any single study, is the principal weakness in the current assessment of methanol’s marine environmental risk.

### 7.5. Bioaccumulation and Persistence: Points of Convergence

Where the literature agrees, it agrees firmly, and the review should say so. The OECD initial assessment reports measured bioconcentration factors below 10 in Cyprinus carpio and Leuciscus idus, consistent with methanol’s low octanol–water partition coefficient [9]. No source reviewed here attributes bioaccumulative potential to it, and impacts are uniformly expected to be short-lived [8,66,74].

It generates no persistent residue, no shoreline oiling, no sediment reservoir and no chronic exposure pathway through the food web—the four mechanisms responsible for the long-term damage characteristic of hydrocarbon spills. Its behavior class, that of a substance that dissolves and evaporates, precludes containment but equally precludes persistence [8,13]. This convergence is substantive and survives the critique advanced above: whatever the true clearance time, methanol does not accumulate.

### 7.6. Receptor-Specific and Habitat-Specific Vulnerability

Risk is a function of where a release occurs, not merely of what is released. Open-ocean events are governed by dilution and are unlikely to generate ecologically meaningful exposures. Enclosed and semi-enclosed settings—bunkering berths, marinas, fjords, lagoons and estuaries—combine restricted exchange with high biological value and concentrate every mechanism identified above. The conditions that concentrate these mechanisms are geographic rather than chemical. Where tidal range is small, the mixing energy that the oil-spill dispersion models of Section 5.1 assume is not available; where seasonal stratification isolates bottom waters, the oxygen demand of roughly 190 m^3^ of saturated seawater per kilogram released is not ventilated; and where a bunkering location adjoins seagrass meadows or finfish aquaculture, the receptors are sessile or caged.

No methanol exposure study has been conducted on either receptor type, and no source located for this review maps bunkering locations against receiving-water conditions of this kind. Sessile benthic communities cannot avoid an oxygen deficit, and caged stock cannot avoid anything at all. Aquaculture exposure is additionally the pathway by which an ecological event becomes an economic and food-safety event, yet it is absent from the risk frameworks currently applied to alternative marine fuels [81,110].

### 7.7. Synthesis

The evidence supports a qualified rather than an unqualified reassurance. Methanol is genuinely benign relative to heavy fuel oil, and on the available evidence to ammonia, on the axes of persistence, bioaccumulation and direct acute toxicity, and the conclusions of earlier comparative work [2,30,111] are not overturned by the present analysis. What the analysis does establish is that the reassurance has been resting on the wrong foundations: on freshwater lethality data from the 1980s, on a half-life imported from ambient biogeochemistry, and on a hypoxia, mechanism acknowledged in prose but never modeled. The residual risk is concentrated, spatially in confined and stratified waters, temporally in the first hours after release, and mechanistically in oxygen depletion rather than in intrinsic toxicity. Whether the ports where methanol bunkering is being established share these conditions is not established in any source located here and is the question the preceding gaps make answerable.

## 8. Risk-Assessment Practice and the Points of Control: Where Toxicity Falls Between the Instruments

### 8.1. Three Assessment Axes and the Hazard None of Them Carries

The question in this section is toxicological rather than legal: whether the hazard characterized in Section 4, Section 5, Section 6 and Section 7 reaches the points at which exposure is actually controlled. Methanol reaches those points along three axes built separately and still not in conversation. The safety axis runs from SOLAS chapter II-1 part G through the IGF Code, as amended [112,113], and, for alcohols, through MSC.1/Circ.1621 [16]. The pollution axis runs from MARPOL Annex II, as amended [78], through the IBC Code [79,80], with hazard classification supplied by the GESAMP procedure [28,69]. The occupational-health axis rests on Regulation 4.3 and Standard A4.3 of the Maritime Labor Convention [114] and, beyond that, on national occupational exposure limits that are not maritime instruments at all. Each axis is internally coherent. What the recent literature contests is whether any of them owns the toxicity of methanol; the emerging answer is that none owns it completely.

On the safety axis, the diagnosis is now convergent. Lee et al. (2026) [3], comparing LNG, LPG, methanol and ammonia against the IGF Code and its interim guidelines, characterize the framework as goal-based but flammability-centered and identify a disconnection between the dominant risk the framework itself acknowledges and the logic by which its safety systems are triggered. Jang et al. (2023) [115] reached a structurally identical conclusion for ammonia two years earlier, on the ground that design criteria built around LNG do not accommodate fuel-specific risk profiles. Ismail et al. (2025) [17] reach the same place from the opposite direction, working from thirty accident investigations rather than from the instruments: their gap analysis sets the barriers implied by the accident record against what MSC.1/Circ.1621 [16] actually requires, and finds those requirements stated in general terms precisely where technical specification is needed, with personal protective equipment being the clearest case. The broader reviews diverge not in direction but in resolution: Hoang et al. (2026) [116] treat the framework as an adoption barrier without disaggregating flammability from toxicity within it, and neither Ceylan (2025) [117] nor Duong et al. (2024) [118] admits a toxicity-specific criterion into the bunkering risk analyses.

On the third axis, the difficulty differs in kind. Standard A4.3, paragraph 4, provides that compliance with applicable international instruments on acceptable levels of exposure to workplace hazards on board ship is to be considered as meeting the Convention [114]. It defers rather than specifies. Ismail et al. (2025) [17] separately establish that MSC.1/Circ.1621 [16] sets no threshold limit value and no toxicity-based alarm. Neither source connects to the other, and the combination is not offered here as a finding but recorded as a gap: no analysis appears to have tested whether the referral in Standard A4.3 has an addressee where methanol is used as fuel rather than carried as cargo.

### 8.2. Classification Worked—But on One Axis Only

The IBC Code entry for methyl alcohol was revised between the 2012 and 2019 amendment cycles, the later text applying from 1 January 2021 [79,80]. Read against each other, the two chapter 17 rows differ in a single family of fields: the hazard designation governing ship type moved from pollution-only to safety-and-pollution, the tank environmental control and gauging entries were tightened, the fire-hazard designation acquired its toxicity component, and the special-requirements column activated the 15.12 provisions for toxic products together with 15.17 and 15.19. The pollution category (Y) and the ship type (3) are unchanged. Every altered field addresses human toxicity; no altered field addresses environmental fate.

This is the strongest available demonstration that the classification machinery is functional rather than inert: it was exercised deliberately, recently, and on exactly this substance—but only along the safety axis. Lee et al. (2026) [3] state the corresponding demand from the other side, calling explicitly for environmental impact assessment criteria for marine leakage and contaminated discharge of toxic fuels—precisely the criteria the 2019 revision left untouched. A scope question compounds this: Annex II governs noxious liquid substances carried in bulk as cargo [78], so a release from the fuel system of a methanol-fueled ship is not an Annex II discharge event, and the pollution category assigned to methanol attaches to a carriage regime the fuel case never enters.

A smaller instance of the same transfer appears inside the regulatory literature itself. A 2025 review of technical standards for clean alternative marine fuels attributes to a comparative life cycle assessment the proposition that spill risk must be considered for fuels not covered by MARPOL Annexes I or II [119]; the study cited is a well-to-propeller assessment of seventeen fuel pathways and advances no such proposition [120]. The sentence appears instead in the regulatory mapping maintained under the IMO GreenVoyage2050 program [121], a resource updated continuously and carrying no version identifier, so the state consulted is that of August 2026. The misattribution matters less than its direction: the observation that the fuel case falls outside the carriage regime—the scope gap set out above—travels onward detached from the institutional source that made it, and therefore from the qualifications that source attaches to it.

### 8.3. Oxygen Demand Counted as Evidence of Harmlessness

The hazard profile feeding Annex II is generated by the GESAMP procedure [28,69], and two of its criteria, read against each other, produce the anomaly developed in Section 5. Column E2, the only column addressing behavior in the marine environment, is evaluated on solubility, melting point, vapor pressure, specific gravity and viscosity—essentially whether the substance forms a slick or coats the seabed. Column A2, biodegradation, is a binary R/NR flag whose criterion for “readily biodegradable” is consumption of at least 60% of the theoretical oxygen demand within 28 days.

Oxygen consumption therefore enters the scheme as evidence that a substance is benign and never re-enters it as a hazard; biochemical oxygen demand is not an endpoint. For methanol, the omission is not academic: with a theoretical oxygen demand of 1.50 kg O_2_ per kilogram and roughly 8 mg/L dissolved oxygen in saturated seawater, one kilogram can stoichiometrically strip some 190 m^3^ of receiving water. This compares two criteria of a single document rather than introducing an analytical separation here, and that document adds a second internal caution: at §4.1.2, it states that biodegradation proceeds more slowly in seawater than in fresh water and prefers marine test protocols to the OECD 301 series—a qualification absent, as Section 5 showed, from every downstream restatement of the aquatic half-life.

### 8.4. Flammability-Anchored Triggers Against a Toxicity-Dominant Hazard

MSC.1/Circ.1621 [16] states its detection logic in numbers: an audible and visible alarm at 20% of the lower explosion limit, and activation of the safety system at 40% of LEL on two detectors [16]. The only toxicity provision attached is the instruction that special consideration should be given to toxicity in designing the detection system, which is a duty without a metric. Ismail et al. (2025) [17] and Lee et al. (2026) [3] document the arithmetic consequence independently of one another: with a lower explosion limit reported between 55,000 and 60,000 ppm, the alarm threshold falls between roughly 11,000 and 12,000 ppm, whereas the NIOSH immediately dangerous to life or health value is 6000 ppm [54,55] and the eight-hour AEGL-3 is 1600 ppm [42]. Concentrations lethal on the timescale of an enclosed-space entry therefore accumulate below the first alarm—a sequence with a clinical counterpart in the bilateral blindness of a seafarer exposed during a wall-wash operation aboard a chemical carrier [5], and a physical one in the dense vapor layer filling a machinery-space domain within seconds of a large-bore release [19].

Nor is there a sensory fallback, and the reason is that the threshold itself is not an established quantity. The European bunkering assessment reports detection from a few parts per million to 5960 ppm, the upper bound effectively coinciding with the immediately dangerous concentration, and states that a person detecting the odor may already have sustained an acute exposure at that level [59], the NIOSH card, cited in Section 6.5, gives a narrower interval of roughly 100 to 1500 ppm [87]. The two do not agree, and the disagreement itself is documented. The committee responsible for the acute exposure guideline levels reached the same position from the same direction: it examined six published threshold series spanning more than three orders of magnitude and concluded that the odor threshold data were not appropriate for deriving its lowest tier at all [42]. Neither the instrument nor the nose is anchored to a concentration at which the hazard begins.

The decisive comparison is with an existing instrument, not a hypothetical one. MSC.1/Circ.1687 [18], approved for ammonia and issued in February 2025, is drafted on the same goal-based template and against the same equivalence standard, yet sets its triggers in parts per million: local visual indication at the entrances to affected enclosed spaces at 25 ppm, audible and visible alarm at 110 ppm, and activation of the safety system at 220 ppm on two detectors [18]. It also does three things the alcohol guidelines do not attempt: it defines toxic areas and toxic spaces as classified categories alongside hazardous areas and requires a dispersion analysis showing that 220 ppm does not reach accommodation intakes, control stations or other non-toxic spaces; it requires a release mitigation system able to reduce the concentration below 110 ppm; and it devotes a full chapter to personnel protection, prescribing decontamination showers at named locations, escape respiratory protection for every person on board and a safe haven sized for all on board.

The point is not that concentration-anchored triggers are unavailable to the regulator: they have been drafted, agreed and issued—four years after MSC.1/Circ.1621 [16], and for the fuel whose toxicity is acute rather than latent. The two hazards are genuinely unlike, the occupational limit for methanol being 200 ppm against 25 ppm for ammonia and its IDLH twenty times higher [3,54,55], so nothing here requires identical treatment. The asymmetry does not track the sensory margin either. Ammonia is detectable by odor at 2 to 5 ppm, two orders of magnitude below its immediately dangerous concentration of 300 ppm, so a leak announces itself long before it disables—although the agency study cautions that odor is not a reliable barrier on its own [122].

For methanol the corresponding margin does not exist. The fuel whose release the crew can smell received toxic-area classification, dispersion analysis and decontamination provision; the fuel whose release they cannot receive a trigger set as a fraction of the lower explosion limit. What the comparison establishes is narrower and harder to dismiss: the absence of any concentration-based toxicity trigger for methanol reflects a drafting choice made within a demonstrated capability, not a limitation of the instrument type.

The asymmetry propagates into the modeling literature: Liu et al. (2024) [89] drive ammonia ship-to-ship bunkering simulations directly onto toxic thresholds and report lethal deck zones, whereas the corresponding methanol work of Khan et al. (2026) [85] resolves individual and societal risk from thermal effects. The ammonia literature is by now dense with toxic-zone determinations of exactly the kind methanol lacks [123,124,125,126].

### 8.5. When the Assessment Gaps Are Scheduled to Close

Both circulars are provisional and explicitly experience-driven: MSC.1/Circ.1621 records that the Committee agreed to keep the guidelines under review in the light of operational experience [16], and MSC.1/Circ.1687 invites Member States to recount their experience to the Organization [18]. Neither specifies what is to be recorded, by whom, or against which reference values. Ismail et al. (2025) [17] supply the practical consequence: of the thirty methanol incidents available to them, twenty-four originated in a shore-based occupational database, and only three in the casualty system of the IMO itself.

Until the guidelines become mandatory, fuels outside the IGF Code depend on the alternative-design route of SOLAS regulation II-1/55, elaborated in MSC.1/Circ.1212/Rev.2 [127]. Lee et al. (2026) [3] argue that absent defined criteria for the scope of approval and the depth of risk assessment, interpretation will diverge between flag states and classification societies, and that alternative design should become a harmonized transitional pathway rather than an exceptional mechanism. Their reading of the CCC and MSC work program for 2024–2027 is that codification is proceeding incrementally, and the record bears this out: the eleventh session of the Sub-Committee, in September 2025, did not reach the revision of MSC.1/Circ.1621 [16] at all.

That revision is now expected in 2027, with the associated mandatory IGF Code amendments targeted for conclusion in 2027 and entry into force in 2028 [61,62,63]. The fleet does not wait on that timetable: fifty-nine methanol dual-fuel ships were in service and 369 on order as of May 2025, and the inspection guidance written for them builds its exposure reasoning from occupational sources, not from any maritime instrument [1], parallel agency study programmes have meanwhile delivered multi-part safety assessments for ammonia and hydrogen [122].

### 8.6. Synthesis: Four Gaps, Stated as Gaps

Four conclusions follow, each being registered as a gap in the record rather than as an original analytical separation. First, no instrument on any of the three axes assigns a numerical exposure criterion to methanol used as a marine fuel: the safety axis declines to set one, the pollution axis does not address human exposure, and the occupational axis defers to instruments that do not supply one—although the dose–response and dermal-uptake literature that would populate such a criterion has existed for decades [41,52]. Second, criteria for third parties and the public exist and are distinguished from occupational limits by their own publisher, which states that the latter protect healthy adult workers across a working lifetime while emergency response planning guidelines protect the public against rare, unexpected, short-term releases [99]. No bunkering or safety-zone instrument reviewed here invokes them. Third, the environmental impact assessment criteria for fuel-system releases requested by Lee et al. (2026) [3] have not been drafted, and Annex II does not reach them because the fuel case falls outside its carriage scope [78]. Fourth, oxygen depletion—identified in Section 5 as the dominant plausible mechanism of ecological harm at spill-relevant mass loadings, and demonstrated for the analogous case of ethanol—has no place in the hazard profile governing the environmental classification of methanol [28,69].

None of this contradicts the assessment that methanol remains the milder option among the leading alternative fuels: the physicochemical comparison supporting that conclusion [2] is untouched by anything above. The argument concerns placement, not magnitude. The instruments are not silent about methanol; each is articulate about a different methanol—a flammable liquid on one axis, a categorized cargo on the second, an unnamed workplace hazard on the third—while the seafarer standing in a machinery space during a leak meets all three at once.

## 9. Discussion

### 9.1. Synthesis: Three Independent Structural Patterns

Section 3, Section 4, Section 5, Section 6, Section 7 and Section 8 did not converge on a single deficiency in the literature on methanol as a marine fuel. They converged on three, and the three are logically independent: each would survive the removal of the other two.

Nine quantitative claims were carried back to the document in which each is generated. Table 3 records the outcome. Two reached the maritime literature with their conditions intact and are reported here as sound; two rest on primary records that could not be retrieved and are flagged rather than adjudicated; the remaining five exhibit one of the three patterns set out below. The proportion is not offered as an estimate of the state of the literature, for the reason given in Section 2.2: these claims were selected because they carry the appraisal of methanol, not sampled at random. What the register does establish is that the finding is not a general suspicion of the corpus. Where a claim was traced and found intact, that is recorded as plainly as the failures.

(A) Saturation of the descriptive vocabulary. Section 4 documented a clinical elimination window for methanol in humans of roughly 2.5 to 87 h [4], with the spread reflecting dose, ethanol co-exposure and antidotal treatment. Section 5 documented an environmental half-life in surface water of one to seven days [8]. The two windows describe different compartments, organisms and processes. Both are nevertheless reported downstream as the same reassurance, namely that methanol does not persist: a range whose upper bound exceeds its lower bound by more than an order of magnitude is compressed into a single adjective, identically in both literatures. The finding is not that either range is wrong. It is that two ranges carrying quite different evidentiary weight are treated as interchangeable grounds for the same conclusion.

(B) Transmission without the method. The second pattern concerns what survives citation and what does not. Three bodies publish three flash points for the same substance: 10 degrees Celsius in the OECD SIDS assessment [9], attributed there explicitly to a closed-cup determination, 11 degrees Celsius in ISO 6583:2024 [26], the normative standard for methanol as a marine fuel; and 12 degrees Celsius in the ITOPF fate-and-behavior report [8], reproduced without qualification in the 2025 response guide of the USCG Research and Development Center [12,13,14]. The spread is operationally trivial, which is what makes it diagnostic: the determination method traveled with the value in 2004 and had been shed by 2024. The same mechanism operates where the stakes are higher. The one-to-seven-day persistence figure originates in the 1991 compilation of degradation rate constants [10], whose surface-water entry is expressly derived from unacclimated aqueous aerobic assays, and reaches the response literature by two routes rather than one: the 1999 Malcolm Pirnie evaluation prepared for the American Methanol Institute [11] tabulates it with the qualifier attached, while the 2024 ITOPF report [8] cites the 1991 compilation directly. The two converge downstream in the 2025 NOAA background sheet [12] and the 2025 USCG guide [12,13,14], and the figure’s apparent corroboration is an artifact of that convergence. The chain is stated rather than inferred: NOAA records that its alternative-fuel sheets are reviewed by ITOPF [8,12], and the USCG guide that it draws on the 2021 Oak Ridge assessment [13] and the 2024 ITOPF series [8,12,13,14]. A value derived from unacclimated aqueous aerobic assays now appears in a United States federal response document as a property of methanol in seawater, stripped of its boundary conditions.

(C) The same property, opposite conclusions. The third pattern is the most consequential, because it cannot be explained by careless copying: three independent bodies have used a single physicochemical property to support incompatible conclusions. The OECD SIDS assessment [9] invokes the low Henry’s law constant of methanol in one section to conclude that volatilization is not a significant removal process from the aquatic compartment, that is, that methanol is retained in the water column, and in another to justify accepting acute LC50 values from unsealed test vessels on the ground that nominal and measured concentrations may be equated. That second use feeds directly into a low-hazard classification and a recommendation of low priority for further work. The GESAMP hazard-evaluation procedure [28,69] exhibits the same structure, as recorded in Section 7; the two bodies are institutionally unrelated. The pattern remains live: a 2026 computational study of methanol-diesel dual-fuel combustion [15] treats full miscibility as evidence that releases dilute rapidly and thereby minimize environmental harm, while the 2025 USCG guide [12,13,14] derives from the identical property the conclusion that mechanical recovery is impossible and that short-term effects in enclosed or low-energy waters are plausible. Neither cites the other. The criticism is not that these bodies reasoned invalidly within their own frames, but that no published source brings the two readings of miscibility into contact, so the incompatibility is never adjudicated.

### 9.2. The Conditions That Bound the Comparative Claim

The comparative judgment of the 2024 review that this paper extends [2] is not disturbed by anything above. On the evidence assembled across Section 4, Section 5, Section 6 and Section 7, methanol remains the milder option relative to heavy fuel oil and to ammonia across the endpoints examined: no persistent residue and no shoreline oiling; measured bioconcentration factors in Cyprinus carpio and Leuciscus idus below 10; acute fish LC50 values between 15,400 and 29,400 mg/L; a 48-h EC50 for Daphnia magna above 10,000 mg/L; and ready biodegradability at 76 to 82 percent within five days [9]. What Section 4, Section 5, Section 6 and Section 7 add is not a revision of that ranking but a specification of the conditions on which it depends, and which the literature carrying it forward rarely states. Four emerge.

First, time to antidote. The severity of human methanol poisoning is governed less by the absorbed dose than by the latency between exposure and the administration of fomepizole or ethanol. At sea, that latency is a function of voyage geography, not of the fuel. No shipboard risk assessment reviewed here treats distance from definitive care as a determinant of methanol hazard.

Second, the phase of the release. Vapor-phase and liquid-phase exposures follow different kinetics, reach different target tissues and call for different protective measures. Aggregating them, as most secondary sources do, discards the distinction on which shipboard controls would have to be built.

Third, the mixing energy of the receiving water. Every dilution-based reassurance in this literature presupposes an open and energetic receiving environment. Dilution is a property of receiving water, not of the fuel. The assumption fails in a closed dock basin, at a semi-enclosed bunkering berth and in an ice-covered channel, which are the settings in which bunkering occurs.

Fourth, enclosed-space geometry. Methanol vapor is denser than air, with a measured ratio of approximately 1.11 [3]. The ITOPF report [8] states explicitly that this density prevents all but a small fraction of a vapor cloud from rising, and that the resulting flammability footprint exceeds that of LNG or hydrogen. The 2025 NOAA background sheet [12] reproduces that reading and records ITOPF review of its alternative-fuel sheets, so the two are not independent statements of it. The 2021 Oak Ridge assessment [13] describes the vapors as rising and dispersing rapidly. The two descriptions are not strictly incompatible: the Oak Ridge statement attributes rapid dispersal to diffusivity in open air, whereas the density argument concerns accumulation in a confined volume, and the computational result [19] bears only on the second. No located source brings the two readings into contact.

### 9.3. Research Gaps as Declared in Published Sources

Section 5, Section 6 and Section 8 record thirteen deficiencies between them. The test applied here is stricter than the one applied at section level: a gap is claimed only where a published source declares it, or where the absence is demonstrable from the corpus. Eight qualify.

(i) Marine fate and transport. The 1999 Malcolm Pirnie evaluation [11] identified the absence of marine-specific fate data as a limitation of its own predominantly freshwater conclusions. Twenty-seven years later, that limitation stands, and the same document remains the upstream citation for figures still in circulation.
(ii) Water toxicity following an incident. The 2025 USCG Research and Development Center guide [12,13,14] records that responders from the USCG Northwest District are concerned by the lack of knowledge about water toxicity resulting from methanol incidents, and that further research is needed.
(iii) Environmental assessment criteria for alternative-fuel leakage. Lee et al. (2026) [3], having documented the flammability-versus-toxicity mismatch on the safety side, call expressly for environmental impact assessment criteria covering marine leakage of alternative fuels. No instrument reviewed here contains such a criterion.
(iv) Aggregation of exposure routes. No published source combines inhalation, dermal and ocular uptake into a single internal dose for a defined shipboard task, although each route is separately characterized in Section 6.
(v) Bunkering frequency as exposure. No source converts bunkering frequency into an expected annual exposure per crew role; the 2025 life-cycle assessment of methanol in dual-fuel engines [30] quantifies only the trade-off between fuel volume and cargo capacity.
(vi) An integrated fuel-comparison matrix. No publication assembles volumetric energy density, occupational exposure limits and marine environmental endpoints for one set of fuels; Table 1 of Section 3 was built because none existed.
(vii) Unreconciled property series. The divergence between the SGMF [31] and the peer-reviewed [3] volumetric energy densities for hydrogen, 9 and 5 MJ/L against 3.4 to 6.8 MJ/L, is unreconciled in any source reproducing either series, though those sources agree closely for methanol, ammonia and LNG.
(viii) The incident record itself. Of the thirty methanol incidents assembled by Ismail et al. (2025) [17], twenty-four came from a shore-based occupational database and only three from the casualty system of the Organization. The evidence base for maritime methanol exposure is therefore built largely outside the maritime reporting system.

These eight are not of equal weight, and the review is more useful if it says so. Ranked by the consequence of leaving them open, three are first-order. Gaps (i) and (ii), marine fate and water toxicity following an incident, are the same deficiency seen from the scientific and the response side, and together, they determine whether any quantitative statement about a methanol release into seawater is possible at all; the coupled release, degradation and dissolved-oxygen simulation for an enclosed or seasonally stratified basin identified in Section 5.4 and Section 7.3 is the single piece of work that would close most of both, and it is tractable with eutrophication modeling infrastructure that already exists. Standing with them is the absence of acute and chronic determinations generated in seawater to a current test guideline, which is not listed separately above only because it is the substance of those two gaps: one modern acute determination on a marine bivalve and one seawater species-sensitivity distribution would replace a single 1996 value that no reader can inspect and would supply the endpoints the simulation needs. Third, and first-order for human safety rather than for the environment, is gap (iv), the aggregation of exposure routes, which is the only one of the three requiring no new experiment: the frameworks named in Section 6.4 need parameterizing with shipboard contact areas, task durations and vapor concentrations.

The remaining gaps are second-order in the sense that closing them improves the evidence base without changing what a risk assessment can presently conclude. Gaps (iii) and (v), environmental assessment criteria for alternative-fuel leakage and bunkering frequency expressed as exposure, are consequential but derivative: they become answerable once the first three are closed, and criteria drafted before then would codify the parameters this review shows to be unsupported. Gaps (vi) and (vii), the integrated fuel-comparison matrix and the unreconciled volumetric energy-density series, are matters of synthesis and editorial reconciliation rather than of measurement and can be closed at negligible cost. Gap (viii), the incident record, is the slowest to close and the least dependent on funding, requiring instead that methanol exposure events be reported into the casualty system of the Organization rather than only into shore-based occupational databases. If one program were to be funded, it should be the coupled hypoxia simulation, together with the seawater toxicity determinations that supply its endpoints; if a second, the route-resolved shipboard dose model, which is the least costly of the three and the most immediately usable by operators.

### 9.4. Implications for Exposure Control, Drawn from Existing Precedent

No original regulatory proposal follows from a narrative review, and none is offered. Section 8 established the decisive comparison from the instruments themselves, and a single arithmetic consequence carries the discussion. MSC.1/Circ.1621 [16] anchors its detection logic to the lower explosion limit: alarm at 20 percent of that limit, safety-system activation at 40 percent on two detectors. With a lower explosion limit reported between 55,000 and 60,000 ppm, the first alarm falls between roughly 11,000 and 12,000 ppm, while the value immediately dangerous to life or health is 6000 ppm [54,55] and the eight-hour AEGL-3 is 1600 ppm [42]. Concentrations capable of blinding or killing a seafarer during an enclosed-space entry accumulate entirely below the threshold at which the ship is told anything at all.

That this is a drafting choice rather than a limit of the instrument type is settled by MSC.1/Circ.1687 [18], written four years later on the same goal-based template and against the same equivalence standard, which sets its triggers in parts per million and adds toxic-area classification, a dispersion criterion, release mitigation and a chapter on personnel protection. The two hazards are genuinely unlike, and nothing here requires identical treatment. What the comparison establishes is narrower: the capability was demonstrated and then not applied. Lee et al. (2026) [3] reach the same conclusion from the instruments, reporting that extending a flammability-centered framework to a fuel whose dominant hazard is toxicity produces a structural mismatch between the hazard and the regulatory trigger.

### 9.5. What Would Overturn These Findings

Each of the three patterns is stated in a form that can be defeated by evidence, and the evidence that would defeat it is specified here so that the claims are not left unfalsifiable. Pattern A, saturation of the descriptive vocabulary, would fail if a document were produced in which the 2.5-to-87-h clinical elimination window and the one-to-seven-day environmental half-life are distinguished at the point of use and the distinction is carried into the reassurance drawn from them.

Pattern B, transmission without the method, would fail for the aqueous half-life if the 1999 Malcolm Pirnie evaluation or the 2024 ITOPF report were shown to have restated the unacclimated-assay conditions, or if a single measured half-life for methanol in seawater were located; a single such determination would convert the apparent corroboration into real corroboration and would remove the principal example on which the pattern rests. Pattern C, one property supporting opposite conclusions, would fail if a published source were located that brings the 2026 combustion study and the 2025 response guide into contact on miscibility, or the 2021 Oak Ridge assessment and the 2024 ITOPF report on vapor density, and adjudicates between them. The three are defeated separately: the failure of any one leaves the other two standing, which is the sense in which they are described here as independent.

### 9.6. Limitations

The form of the review sets the limits of what it can establish. Section 2.1 gives the reason for adopting the narrative form; the cost is that no protocol was registered and the search, although directed by the claims examined and documented as to procedure in Section 2.3, was not exhaustive. No standardized risk-of-bias instrument was applied, with none of the available instruments being designed for documents whose function is transmission rather than measurement. What was done in their place is set out in Section 2.2 and recorded in Table 3: the separation of originating, transmitting and adjudicating functions, the identification of commissioning interest where the document declares it, and the flagging of primary records that could not be opened. The review generates no primary data and cannot therefore close any of the gaps it identifies. What it can establish is that they are open.

Selection was purposive, directed at documents that carry, transmit or adjudicate the claims examined. It was not, however, discretionary at the point on which the argument turns. Once a terminal claim is fixed, the documents upstream of it are determined by the citations themselves rather than chosen by the reviewer, so every chain reconstructed in Section 5 and Section 9 can be walked again by any reader and either confirmed or broken at a named link. What a different reviewer could vary is the starting set of claims, and a different starting set would surface different chains. It would not alter the provenance of the nine examined here, which is a property of the documents and not of this review. The same distinction governs what incompleteness can do to the result. Exhaustive coverage is required where the claim is one of prevalence, since an unsampled remainder can move a proportion in either direction.

The claims made here are of existence: that a stated qualifier is present in one document and absent from the document that cites it. A claim of that form is true or false of the two documents named, and no quantity of further searching can falsify it. What further searching could do is add chains that went unreported, so incompleteness in the corpus tends to understate the extent of transmission failure rather than to exaggerate it. Misreading is a different risk, met by a different control: every record was verified against the issuing body’s own version of the item, and every chain is stated in a form the reader can walk.

The age distribution of the corpus requires the same distinction. About three-fifths of the bibliographic sources fall within the five years preceding the close of searching, and some two dozen predate 2010; that older material is not residual but is the set of primary determinations and compilations in which the claims examined originate, for the reason set out in Section 2.4. The constraint that does follow is narrower, and is real: where a claim originates in a document that is both old and not publicly retrievable, the audit stops at the last accessible link, and the origin is reported as reached rather than read.

That constraint binds at several points, notably the 1999 fate-and-transport work and the pre-2000 acute toxicity determinations, where the primary records were not publicly retrievable during preparation, and the review relies on secondary reproduction. The reliance is of the same kind as the one examined in Section 5 and Section 9, and differs in the respect on which that examination turns: here, the route is declared where the value is used, and the reader is told which link could not be opened, whereas the documents examined there present a transmitted value as a property of the substance. One instance bears directly on the argument. The ecotoxicological assessment in Section 7 rests, for the marine compartment, on a single acute determination whose primary record was not retrievable, reaching this review through a secondary account; the remaining acute data are freshwater. That the argument is thinnest at exactly this point is stated rather than concealed, and the thinness is itself the clearest available statement of the gap identified: the marine acute toxicity of a fuel now entering service is represented in the accessible literature by one determination made thirty years ago.

## 10. Conclusions

Methanol remains the milder of the marine fuels now in serious contention. Against heavy fuel oil, it leaves no persistent residue and no oiled shoreline, and measured bioconcentration factors lie below 10 [9]. Against ammonia, the vapor thresholds differ in methanol’s favor by roughly an order of magnitude—an eight-hour occupational limit of 200 ppm against 25 ppm, and a value immediately dangerous to life or health twenty times higher [3,54,55]—while no source located for this review measures the aquatic toxicity of the two fuels on a common basis. The review therefore ends where the baseline assessment began. It ends there for better-examined reasons.

What changes is the standing of the ranking, not its direction. It has traveled through the literature as a property of the substance, when it is a property of the substance under conditions that are seldom stated. Four conditions bound it: the interval between exposure and antidote, which at sea is set by voyage geography and not by the fuel; the phase in which the release occurs, vapor and liquid following different kinetics to different tissues; the mixing energy of the receiving water, since dilution is a property of the water and not of the fuel; and the geometry of the space in which vapor collects, with methanol vapor being denser than air and slow to rise. Stated with those conditions attached, the comparative claim is defensible. Stated without them, it is an assertion.

Three structural patterns explain how the conditions came to be dropped. A descriptive vocabulary compresses ranges spanning more than an order of magnitude into a single reassuring adjective and does so identically in the clinical and the environmental literatures. Values travel intact along chains of citation while the methods that produced them fall away, so that a figure generated for one largely freshwater study in the 1990s now appears in federal response guidance stripped of its boundary conditions. And a single physical property is read by separate authorities to opposite conclusions, none of which meets the others, so the incompatibility is never adjudicated. None of these is a data gap in the ordinary sense, and none is closed by producing more data.

One distinction should be drawn explicitly, because the argument is easily over-read. What this review audits is transmission, not measurement. The primary determinations examined here were, with two exceptions, competently made, and are reported by their authors with the conditions under which they hold: the 1991 compilation states that its surface-water figure derives from unacclimated aqueous aerobic assays, the intergovernmental assessment states that its flash point is a closed-cup determination, and the nanomolar uptake rates were measured as such and described as such. What fails is downstream, where a secondary or industry document reproduces the value without the condition, and a later document reads the unconditioned value as a property of the substance. The two exceptions are of a different kind and are marked as such wherever they are used: the 1996 marine acute determination and the 1987 and 1989 dose reports, whose primary records could not be retrieved, where the limitation lies in what this review could verify and not in what was originally done. Nothing here should be read as a finding that the underlying toxicological experiments are widely defective. The finding is that conditions attached to sound results do not survive the route by which those results reach the people who act on them.

The regulatory consequence is the sharpest of them. Detection aboard methanol-fueled ships is anchored to flammability, with the result that concentrations capable of blinding or killing a seafarer during an enclosed-space entry are reached well before the first alarm sounds. This is not a limitation of the instrument type. A concentration-based architecture for a toxic marine fuel has already been drafted, agreed and issued for a different fuel, on the same template and against the same equivalence standard. The elements exist; they have not been carried across, and no published record shows the question having been put.

The case for methanol does not require the confidence with which its fate and exposure parameters are presently stated. It requires the opposite. The parameters should be reported with the conditions that bound them; the gaps identified here should be described as gaps rather than absorbed into reassurance; the eight should be addressed in the order of consequence set out in Section 9.3, beginning with the coupled release, degradation and dissolved-oxygen simulation for an enclosed basin and the seawater toxicity determinations that would supply its endpoints; and the provisions already written for a toxic marine fuel should be either carried across or explicitly declined. Until that happens, the most defensible claim the sector makes about methanol will continue to rest on an evidence base considerably weaker than the claim itself.

## Figures and Tables

**Table 1:** Volumetric energy density and inhalation reference values for candidate marine fuels, as reported in the peer-reviewed comparative appendix of [3], with the independent series of [31] as reproduced in [12,13,14]. Dashes indicate values not stated in the source and are not assertions that no such value exists.

Fuel	Vol. Energy Density (GJ/m^3^) [3]	Independent Series (MJ/L) [14,31]	Inhalation Reference Value (ppm) [3]	Storage Condition
Methanol	15.8	16	200 (TLV–TWA)	Liquid at ambient conditions; miscible with water
Ammonia	11.5	13	25 (TLV–TWA); IDLH 300	Liquefied, −33 °C
LNG	21.5	21	1000	Cryogenic, −162 °C
LPG	26.8	25	—	Pressurized or refrigerated
Hydrogen	3.4–6.8	9 liquid/5 compressed	—	Compressed or liquefied, −253 °C
Marine gas oil	≈36.6	33 (biodiesel, nearest listed)	—	Liquid at ambient conditions; baseline

**Table 2:** Occupational exposure limits for methanol across jurisdictions.

Jurisdiction/Body	Instrument (Year)	8-h TWA	Short-Term Limit	Skin	Emergency/Ceiling Value
NIOSH (US, advisory)	Pocket Guide, CAS 67-56-1	200 ppm (260 mg m^−3^)	250 ppm (325 mg m^−3^)	Yes	IDLH 6000 ppm
OSHA (US, enforceable)	29 CFR 1910.1000 Table Z-1	200 ppm (260 mg m^−3^)	Not in binding form	No	Not set
European Union (indicative)	Directive 2006/15/EC, 2nd IOELV list	200 ppm (260 mg m^−3^)	Not set	Yes	Not set
China (enforceable)	GBZ 2.1-2019	25 mg m^−3^ (PC-TWA)	50 mg m^−3^ (PC-STEL)	Yes	Not set
IMO (international shipping)	MSC.1/Circ.1621 (2020)	No numerical limit	No numerical limit	n/a	Goal-based: segregation, double barriers, detection, isolation

Sources: [16,54,55,56,57,58]. Values current as on the date of compilation. Values are as stated by each body; The ACGIH threshold limit values are not reproduced in this table; where they are referred to in the text, they are cited from the peer-reviewed comparative appendix of [3]. The milligram-per-cubic-meter figures are those published by the issuing authority, not converted here, and the reference conditions differ between instruments. For orientation, the conversion at 20 °C and 101.3 kPa is 1 ppm ≈ 1.33 mg m^−3^, and at 25 °C approximately 1.31 mg m^−3^. The ratio between the Euro-American eight-hour consensus (260 mg m^−3^) and the Chinese permissible concentration (25 mg m^−3^) is approximately 10.4:1.

**Table 3:** Register of the claims traced in this review. Documents are named by function, not by citation number; the corresponding references are given at the point where each claim is examined in Section 3, Section 4, Section 5, Section 6, Section 7 and Section 8. “Degraded” denotes a value that survives transmission while its conditions of measurement do not; “pseudo-convergence” denotes agreement among documents sharing a single origin; “sound” denotes a claim that reached the maritime literature with its conditions intact.

Claim Audited	Where the Value Is Generated	How It Reaches the Maritime Literature	What Is Lost in Transit	Status
Aqueous half-life, 1–7 days	Unacclimated aqueous aerobic assays, compiled 1991	Two routes: a 1999 consultancy evaluation and, independently, the 2024 industry report; converging in two 2025 federal documents	The assay qualifier, and with it the concentration regime to which the rate applies	Degraded
Flash point, 10/11/12 °C	Closed-cup determination reported in the intergovernmental assessment	Normative standard and industry series, then the 2025 response guide	The determination method	Degraded; magnitude trivial
Henry’s law constant	Intergovernmental assessment	Used within the same document to establish retention in the water column and to accept results from unsealed vessels	Nothing is lost; the same value is applied to incompatible ends	Contradictory application
Eight-hour occupational limit, 200 ppm	A single 1968 list	Adopted by four instruments across three jurisdictions	The fact of common ancestry; four statements read as four evaluations	Pseudo-convergence
Vapor-to-air density ratio, 1.11	Measured, and independently modeled by computational fluid dynamics	Described in opposite terms by a national laboratory assessment and by the industry and response literature	No published source brings the two descriptions into contact	Unadjudicated
Marine acute lethal concentration, Mytilus edulis	A single 1996 determination	Reported through a 2004 secondary account	The primary record was not retrievable for this review	Flagged; sole marine datum
Lethal and exposure doses	Two reports of 1987 and 1989	Known here only through later secondary reproduction	Primary records not retrievable	Flagged
Bioconcentration factors below 10	Measured determinations reported in the intergovernmental assessment	Reproduced consistently across the corpus	Nothing; the conditions travel with the value	Sound
Non-persistence and non-bioaccumulation classification	Hazard evaluation procedure of the intergovernmental expert group	Applied consistently by classification and response literature	Nothing	Sound

## Data Availability

All data analyzed in this systematic review were derived from previously published studies, which are cited in the manuscript and listed in the references. No new primary data were generated during this study.

## Supplementary Materials

The following supporting information can be downloaded at https://www.mdpi.com/article/10.3390/toxics14100868/s1, Table S1: Functional classification of the reference list.

## Abbreviations

The following abbreviations are used in this manuscript:

ACGIH	American Conference of Governmental Industrial Hygienists
ADH	Alcohol dehydrogenase
AEGL-3	Acute Exposure Guideline Level 3
A2	GESAMP hazard profile column A2 (biodegradation)
CAS	Chemical Abstracts Service (registry number)
CCC	Sub-Committee on Carriage of Cargoes and Containers (IMO)
CFR	Code of Federal Regulations (United States)
EC50	Median effective concentration
E2	GESAMP hazard profile column E2 (behavior in the marine environment)
GBZ	Guojia Zhiye Weisheng Biaozhun (Chinese national occupational health standard series)
GESAMP	Joint Group of Experts on the Scientific Aspects of Marine Environmental Protection
GJ	Gigajoule
IBC Code	International Code for the Construction and Equipment of Ships Carrying Dangerous Chemicals in Bulk
IDLH	Immediately dangerous to life or health
IGF Code	International Code of Safety for Ships Using Gases or Other Low-flashpoint Fuels
IMO	International Maritime Organization
IOELV	Indicative occupational exposure limit value (European Union)
IPCS	International Program on Chemical Safety
ISO	International Organization for Standardization
ITOPF	International Tanker Owners Pollution Federation
Kow	Octanol–water partition coefficient
LC50	Median lethal concentration
LEL	Lower explosion (explosive) limit
LNG	Liquefied natural gas
LPG	Liquefied petroleum gas
MARPOL	International Convention for the Prevention of Pollution from Ships
MJ	Megajoule
MRI	Magnetic resonance imaging
MSC	Maritime Safety Committee (IMO); MSC.1/Circ. = Maritime Safety Committee circular
NIOSH	National Institute for Occupational Safety and Health (United States)
NOAA	National Oceanic and Atmospheric Administration (United States)
OECD	Organisation for Economic Co-operation and Development
ORNL	Oak Ridge National Laboratory
OSHA	Occupational Safety and Health Administration (United States)
P&I	Protection and indemnity (marine insurance)
PC-STEL	Permissible concentration—short-term exposure limit (China)
PC-TWA	Permissible concentration—time-weighted average (China)
PNEC	Predicted no-effect concentration
PPE	Personal protective equipment
ppm	Parts per million
R/NR	Readily biodegradable/not readily biodegradable (GESAMP binary flag)
RT	Gas constant multiplied by absolute temperature
SGMF	Society for Gas as a Marine Fuel
SIAM	SIDS Initial Assessment Meeting
SIDS	Screening Information Data Set (OECD)
SOLAS	International Convention for the Safety of Life at Sea
T1–T4	Toxicity exposure codes: dermal (T1), ocular (T2), inhalation (T3), ingestion (T4)
TLV–TWA	Threshold limit value—time-weighted average
TWA	Time-weighted average
US	United States
USCG	United States Coast Guard
WHO	World Health Organization
