# Supplementary material for: Human Toxicity and Marine Ecotoxicological Risk of Methanol as a Ship Fuel: A Critical Narrative Review of Exposure, Fate and Aquatic Effects
Source: Toxics. 2026 Sep 29;14(10):868. doi: 10.3390/toxics14100868 (PMC13709557; doi:10.3390/toxics14100868)
Supplement: Supplementary file 1 [file toxics-14-00868-s001.zip › toxics-4572193-supplementary.pdf]

Supplementary Materials

Human Toxicity and Marine Ecotoxicological Risk of Methanol as a Ship Fuel: A Critical Narrative Review of Exposure, Fate and Aquatic Effects

Table S1. Functional classification of the reference list

Each entry in the reference list is assigned to one of the three categories defined in Section 2.4 of the article. An item counts as a bibliographic source when it reports data, an analysis or an argument; as a normative instrument when it prescribes a requirement, procedure, specification or limit value; and as a methodological reference when it is cited only for the conduct of the review. Instruments and methodological references are excluded from the currency figures given in Section 2.4 and Section 9.6, because the currency of an instrument is set by force and amendment status rather than by date of issue.

Applying that test to the 127 entries yields 106 bibliographic sources, 15 normative instruments and 6 methodological references. Every bibliographic source carries a year of issue. Of those, 64 (60 per cent) were published within the five years preceding the close of searching on 16 August 2026, and 23 predate 2010. Entry [121] is a continuously maintained online resource carrying no version identifier; the year given is that of its last recorded revision, and the state consulted is that of August 2026, as stated in Section 8.2.

Two assignments rest on judgement rather than on the explicit wording of Section 2.4. The documentation supporting the acute exposure guideline levels [43] is named there as an instrument; the emergency response planning guideline documentation [99] is classified here on the same basis, and the technical standard [84] is treated as an instrument although it is also the only European specification for the operation it governs. The figures are not sensitive to these choices: reclassifying [43] and [99] as bibliographic sources moves the five-year proportion from 60 to 59 per cent and the pre-2010 count from 23 to 24.

Titles are abbreviated for legibility. Full bibliographic details are given in the References section of the article, in the same numbering. Entries [95] to [98] were added at revision, in support of the new paragraph in Section 6.4 of the article, and are classified as bibliographic sources on the same test; the entries that followed them have been renumbered accordingly.

Ref.	Entry (abbreviated)	Year	Class
1	Maritime Technologies Forum Guidelines for Conducting Safe Inspections of Methanol Dual-Fuel Ships. Maritime…	2025	Source
2	Parris, D.; Spinthiropoulos, K.; Ragazou, K.; Giovou, A.; Tsanaktsidis, C. Methanol, a Plugin Marine Fuel…	2024	Source
3	Lee, J.; Kim, C.; Hwang, D.-J.; An, S.; Kim, M. A Risk-Informed Comparative Analysis of IMO Safety…	2026	Source
4	Alrashed, M.; Aldeghaither, N.S.; Almutairi, S.Y.; Almutairi, M.; Alghamdi, A.; Alqahtani, T.; Almojathel,…	2024	Source
5	Alrougi, H.M.; Saqah, R.; Khojah, I.; Amer, S.A. Occupational Methanol Toxicity: A Case Report Study.…	2025	Source
6	Kaviraj, A.; Bhunia, F.; Saha, N.C. Toxicity of Methanol to Fish, Crustacean, Oligochaete Worm, and Aquatic…	2004	Source
7	Dixon, J.L.; Beale, R.; Nightingale, P.D. Microbial Methanol Uptake in Northeast Atlantic Waters. The ISME…	2011	Source
8	ITOPF Fate, Behaviour, Potential Damage & Liabilities Arising from a Spill of Methanol in the Marine…	2024	Source
9	Organisation for Economic Co-operation and Development (OECD) SIDS Initial Assessment Profile: Methanol (CAS…	2004	Source
10	Howard, P.H.; Boethling, R.S.; Jarvis, W.F.; Meylan, W.M.; Michalenko, E.M. Handbook of Environmental…	1991	Source
11	Malcolm Pirnie, Inc. Evaluation of the Fate and Transport of Methanol in the Environment; American Methanol…	1999	Source
12	(NOAA), Office of Response and Restoration Methanol as a Marine Fuel; U.S. Department of Commerce, National…	2025	Source
13	Kass, M.; Sluder, C.; Kaul, B. Spill Behavior, Detection, and Mitigation for Emerging Nontraditional Marine…	2021	Source
14	U.S. Coast Guard Research and Development Center Appendix F. Biofuels Quick Response Guide; Operational…	2025	Source
15	Pham, V.C.; Le, V.V.; Choi, J.-H.; Lee, W.-J. CFD Analysis of Equivalence Ratio Effects on Combustion and…	2026	Source
16	International Maritime Organization (IMO) MSC.1/Circ.1621 - Interim Guidelines for the Safety of Ships Using…	2020	Instrument
17	Ismail, A.M.; Metwalli, M.M.A.; Alamoush, A.S. Towards Safe Maritime Decarbonization: Safety Barriers of…	2025	Source
18	International Maritime Organization (IMO) MSC.1/Circ.1687- Interim Guidelines for the Safety of Ships Using…	2025	Instrument
19	Sun, B.; Yang, G.; Li, J.; Liu, X.; Sun, Y. Study on the Diffusion Mechanisms of Methanol Leakage in…	2025	Source
20	Greenhalgh, T.; Thorne, S.; Malterud, K. Time to Challenge the Spurious Hierarchy of Systematic over…	2018	Methodological
21	Sukhera, J. Narrative Reviews: Flexible, Rigorous, and Practical. Journal of Graduate Medical Education…	2022	Methodological
22	Greenhalgh, T.; Peacock, R. Effectiveness and Efficiency of Search Methods in Systematic Reviews of Complex…	2005	Methodological
23	Page, M.J.; McKenzie, J.E.; Bossuyt, P.M.; Boutron, I.; Hoffmann, T.C.; Mulrow, C.D.; Shamseer, L.;…	2021	Methodological
24	Adams, R.J.; Smart, P.; Huff, A.S. Shades of Grey: Guidelines for Working with the Grey Literature in…	2017	Methodological
25	Adams, J.; Hillier-Brown, F.C.; Moore, H.J.; Lake, A.A.; Araujo-Soares, V.; White, M.; Summerbell, C.…	2016	Methodological
26	International Organization for Standardization (ISO) Methanol as a Fuel for Marine Applications --- General…	2024	Instrument

Ref.	Entry (abbreviated)	Year	Class
27	Sander, R. Compilation of Henry's Law Constants (Version 5.0.0) for Water as Solvent. Atmos. Chem. Phys.…	2023	Source
28	Joint Group of Experts on the Scientific Aspects of Marine Environmental Protection (GESAMP) The Revised…	2014	Instrument
29	Pham, V.C.; Ngo, D.N.; Hoang, V.S.; Le, V.V. Comprehensive Review of Methanol and LNG as Alternative Fuels…	2026	Source
30	Masum, F.H.; Tan, E.C.D.; Kolodziej, C.P.; Hawkins, T.R. Life Cycle Assessment of Methanol from Fossil,…	2025	Source
31	Society for Gas as a Marine Fuel (SGMF) Methanol as a Marine Fuel --- An Introduction; Society for Gas as a…	2024	Source
32	McMartin, K.; Jacobsen, D.; Hovda, K.E. Antidotes for Poisoning by Alcohols That Form Toxic Metabolites.…	2025	Source
33	Ashurst, J.V.; Schaffer, D.H.; Nappe, T.M. Methanol Toxicity. In StatPearls; StatPearls Publishing: Treasure…	2026	Source
34	Liesivuori, J.; Savolainen, A.H. Methanol and Formic Acid Toxicity: Biochemical Mechanisms. Pharmacology &…	1991	Source
35	WHO/International Programme on Chemical Safety (IPCS) Methanol; Environmental Health Criteria; World Health…	1997	Source
36	Zakharov, S.; Kurcova, I.; Navratil, T.; Salek, T.; Komarc, M.; Pelclova, D. Is the Measurement of Serum…	2015	Source
37	Black, K.A.; Eells, J.T.; Noker, P.E.; Hawtrey, C.A.; Tephly, T.R. Role of Hepatic Tetrahydrofolate in the…	1985	Source
38	Johlin, F.C.; Fortman, C.S.; Nghiem, D.D.; Tephly, T.R. Studies on the Role of Folic Acid and…	1987	Source
39	Zakharov, S.; Nurieva, O.; Navratil, T.; Diblik, P.; Kuthan, P.; Pelclova, D. Acute Methanol Poisonings:…	2014	Source
40	Alnefaie, S.A.; Aldlgan, A.A.; Albakiri, K.M.; Kaabi, M.A.; Alzwen, G.M.; Al-Otaibi, S.S.; Alasmari, F.…	2024	Source
41	Wu, X.; Gu, M.; Wang, W.; Zhang, H.; Tang, Z. Corrigendum: Case Report: Early Recognition, Treatment, and…	2023	Source
42	Batterman, S.A.; Franzblau, A. Time-Resolved Cutaneous Absorption and Permeation Rates of Methanol in Human…	1997	Source
43	National Advisory Committee for Acute Exposure Guideline Levels for Hazardous Substances (NAC/AEGL…	2005	Instrument
44	Wu, X.; Gu, M.; Wang, W.; Zhang, H.; Tang, Z. Case Report: Early Recognition, Treatment, and Occupational…	2022	Source
45	Maritime Just Transition Task Force Considerations of Training Aspects for Seafarers on Ships Powered by…	2024	Source
46	Sharpe, J.A.; Hostovsky, M.; Bilbao, J.M.; Rewcastle, N.B. Methanol Optic Neuropathy: A Histopathological…	1982	Source
47	Da Silva, R.O.; Ohannesian, V.A.; Aguirre, B.N.; Gerente, B.G.D.S.M.; Meira, O.T.; Soares, C.; Palazoni, V.;…	2026	Source
48	Mojica, C.V.; Pasol, E.A.; Dizon, M.L.; Kiat, W.A.; Lim, T.R.U.; Dominguez, J.C.; Valencia, V.V.; Tuaño,…	2020	Source
49	Nagendran, V.; Cabourne, E.; Petzold, A. Methanol Toxicity; Characteristics, Ophthalmological Sequelae and…	2025	Source
50	Zakharov, S.; Pelclova, D.; Diblik, P.; Urban, P.; Kuthan, P.; Nurieva, O.; Kotikova, K.; Navratil, T.;…	2015	Source
51	Ahmadi, S.; Ostadi, A.; Chitsazi, H.; Alikhah, H. Clinical and Laboratory Prognostic Factors Associated with…	2025	Source
52	Machiele, P.A. Flammability and Toxicity Tradeoffs with Methanol Fuels (SAE Technical Paper 872064); SAE…	1987	Source
53	Moon, C.-S. Estimations of the Lethal and Exposure Doses for Representative Methanol Symptoms in Humans. Ann…	2017	Source
54	National Institute for Occupational Safety and Health NIOSH Pocket Guide to Chemical Hazards; U.S.…	2007	Source
55	National Institute for Occupational Safety and Health Documentation for Immediately Dangerous to Life or…	1994	Source
56	European Commission Commission Directive 2006/15/EC of 7 February 2006 Establishing a Second List of…	2006	Instrument
57	Occupational Safety and Health Administration Occupational Safety and Health Standards, Subpart Z, Toxic and…	1971	Instrument
58	National Health Commission of the People's Republic of China Occupational exposure limits for hazardous…	2019	Instrument
59	European Maritime Safety Agency Update on Potential of Biofuels in Shipping; European Maritime Safety…	2022	Source
60	European Maritime Safety Agency Safe Bunkering of Biofuels: Bunkering of Biofuels in Maritime ---…	2023	Source
61	Lloyd's Register CCC 11 Summary Report; Lloyd's Register Group Limited: London, 2025;	2025	Source
62	American Bureau of Shipping CCC 11 Brief; American Bureau of Shipping: Spring, TX, 2025;	2025	Source
63	DNV IMO CCC 11: Interim Guidelines for Hydrogen as Fuel Completed; DNV AS: Høvik, 2025;	2025	Source
64	Hoenders, R. IMO's Ruleset and Policies Relevant for the Use of Biofuels.; International Maritime…	2026	Source
65	Le Masurier, A. Alternative Fuels: A Shift in the Response Paradigm?; ITOPF: London, May 23 2024.	2024	Source
66	Ghosh, A. Methanol - Alternative Fuel Spill Response Considerations - UK P&I; uk p&I Club, 2026;	2026	Source
67	Machiele, P.A. A Perspective on the Flammability, Toxicity, and Environmental Safety Distinctions between…	1989	Source
68	Willey, J.D.; Powell, J.P.; Avery, G.B.; Kieber, R.J.; Mead, R.N. Use of Experimentally Determined Henry's…	2017	Source
69	Joint Group of Experts on the Scientific Aspects of Marine Environmental Protection (GESAMP) GESAMP Hazard…	2019	Instrument
70	Dixon, J.L.; Sargeant, S.; Nightingale, P.D.; Colin Murrell, J. Gradients in Microbial Methanol Uptake:…	2013	Source
71	Butler, B.J.; VanderGriendt, M.R.; Barker, J.F. Impact of Methanol on the Biodegradative Activity of Aquifer…	1992	Source
72	Novak, J.T.; Goldsmith, C.D.; Benoit, R.E.; O'Brien, J.H. Biodegradation of Methanol and Tertiary Butyl…	1985	Source
73	Katsumata, P.T.; Kastenberg, W.E. Fate and Transport of Methanol Fuel from Spills and Leaks. Hazardous Waste…	1996	Source
74	Dinasquet, J.; Tiirola, M.; Azam, F. Enrichment of Bacterioplankton Able to Utilize One-Carbon and…	2018	Source
75	(NOAA), Office of Response and Restoration Denatured Ethanol Spills; Oil Fact Sheets for Spill Responders;…	2019	Source
76	Shaw Environmental and Infrastructure Group Large Volume Ethanol Spills -- Environmental Impacts & Response…	2011	Source
77	Cheng, S.; Meng, F.; Wang, Y.; Zhang, J.; Zhang, L. The Potential Linkage between Sediment Oxygen Demand and…	2024	Source
78	International Maritime Organization Amendments to the Annex of the Protocol of 1978 Relating to the…	2004	Source
79	International Maritime Organization 2012 Amendments to the International Code for the Construction and…	2012	Source
80	International Maritime Organization 2019 Amendments to the International Code for the Construction and…	2019	Source
81	Mahía-Prados, J.M.; Arias-Fernández, I.; Gómez, M.R.; Pereira, S. Analysis About the Leaks and Explosions of…	2026	Source

Ref.	Entry (abbreviated)	Year	Class
82	American Bureau of Shipping Methanol Bunkering: Technical and Operational Advisory; American Bureau of…	2024	Source
83	Indian Register of Shipping Best Practices - Methanol Bunkering, 2025; Indian Register of Shipping: Mumbai,…	2025	Source
84	European Committee for Standardization EN 18071:2025 - Inland Navigation Vessels - Methanol Bunkering; CEN:…	2025	Instrument
85	Khan, H.B.; Zhang, T.; Pan, Z.S. Dynamic Risk Analysis of Bunkering Operations Based on System Dynamics…	2026	Source
86	Kong, X.; Jiao, W.; Xiang, W.; Wang, Q.; Cao, J.; Han, L. Quantitative Analysis of Leakage Consequences of…	2023	Source
87	National Institute for Occupational Safety and Health Methanol: Systemic Agent; U.S. Department of Health…	2003	Source
88	Log, T.; Moi, A.L. Ethanol and Methanol Burn Risks in the Home Environment. IJERPH 2018, 15, 2379,…	2018	Source
89	Liu, Y.; Harikrishnan, B.; Kolluru, R.; Mastorakos, E. Computational Fluid Dynamics Simulation of Ammonia…	2024	Source
90	Hwang, I.; Jeong, B.; Zhou, P.; Wang, H.; Mesbahi, A.; Jang, H.; Mujeeb-Ahmed, M.P.; Yang, I. A PIDA-PDA…	2026	Source
91	Gothenburg Port Authority Methanol Bunker Operating Regulations; Göteborgs Hamn AB (municipal company, City…	2023	Source
92	Shen, H.; Zhong, L.X.; Fu, Y.; Shao, W.; Yang, Y.; Wang, Z.; Yang, H. Factors Affecting the Severity and…	2023	Source
93	Givens, M.; Kalbfleisch, K.; Bryson, S. Comparison of Methanol Exposure Routes Reported to Texas Poison…	2008	Source
94	DeBord, D.G.; Shoemaker, D.; B'Hymer, C.; Snawder, J. Application of Biological Monitoring Methods for…	2022	Source
95	ten Berge, W. A Simple Dermal Absorption Model: Derivation and Application. Chemosphere…	2009	Source
96	Marquart, H.; Franken, R.; Goede, H.; Fransman, W.; Schinkel, J. Validation of the Dermal Exposure…	2017	Source
97	Bouchard, M.; Brunet, R.C.; Droz, P.-O.; Carrier, G. A Biologically Based Dynamic Model for Predicting…	2001	Source
98	Teeguarden, J.G.; Tan, Y.-M.; Edwards, S.W.; Leonard, J.A.; Anderson, K.A.; Corley, R.A.; Kile, M.L.;…	2016	Source
99	AIHA Guideline Foundation Methanol - Emergency Response Planning Guidelines (ERPG); AIHA Guideline…	2017	Instrument
100	Bilgili, L. A Systematic Review on the Acceptance of Alternative Marine Fuels. Renewable and Sustainable…	2023	Source
101	Alamoush, A.S. Trends in Port Decarbonisation Research: Are We Reinventing the Wheel? Current Opinion in…	2024	Source
102	Helmstetter, A.; Gamerdinger, A.P.; Pruell, R.J. Acute Toxicity of Methanol to Mytilus Edulis. Bulletin of…	1996	Source
103	Wissner, N.; Healy, S.; Cames, M.; Sutter, J. Methanol as a Marine Fuel: Advantages and Limitations;…	2023	Source
104	NorthStandard Methanol as a Marine Fuel; NorthStandard Ltd.: Newcastle upon Tyne, 2024;	2024	Source
105	Winterhalter, J. Green Methanol: A Deep Dive into the Alternative Marine Fuel; Port of Seattle: Seattle, WA,…	2025	Source
106	Knightes, C.D. Simulating Hypoxia in a New England Estuary: WASP8 Advanced Eutrophication Module…	2023	Source
107	Glass, J.B.; Warters, L.N.; Alajlan, A.M. Diel Cycle of Lanthanide-Dependent Methylotrophy by…	2025	Source
108	Howat, A.M.; Vollmers, J.; Taubert, M.; Grob, C.; Dixon, J.L.; Todd, J.D.; Chen, Y.; Kaster, A.-K.; Murrell,…	2018	Source
109	Fischer, P.Q.; Sánchez-Andrea, I.; Stams, A.J.M.; Villanueva, L.; Sousa, D.Z. Anaerobic Microbial Methanol…	2021	Source
110	Mahía-Prados, J.M.; Arias-Fernández, I.; Gómez, M.R.; Pereira, S. Hybrid Methodology for Alternative Fuels…	2026	Source
111	Hazar, C.; Seyhan, A. Investigating Ammonia as an Alternative Marine Fuel: A SWOT Analysis Using the…	2025	Source
112	International Maritime Organization Resolution MSC.391(95) - Adoption of the International Code of Safety…	2015	Instrument
113	International Maritime Organization Resolution MSC.392(95) - Amendments to the International Convention for…	2015	Instrument
114	International Labour Organization Maritime Labour Convention, 2006, as Amended; International Labour…	2006	Instrument
115	Jang, H.; Mujeeb-Ahmed, M.P.; Wang, H.; Park, C.; Hwang, I.; Jeong, B.; Zhou, P.; Mickeviciene, R.…	2023	Source
116	Hoang, A.T.; Chen, W.-H.; López-Escalante, M.C.; Guerrero-Pérez, M.O.; Rodríguez-Castellón, E.; Kowalski,…	2026	Source
117	Ceylan, B.O. Ship Bunkering Operations Risk Assessment Using Rule-Based Fuzzy Failure Mode Effect Analysis…	2025	Source
118	Duong, P.A.; Ryu, B.R.; Jung, J.; Kang, H. A Comprehensive Review of the Establishment of Safety Zones and…	2024	Source
119	Wang, S.; Liu, Y.; Zhang, J.; Zhao, L.; Liu, X. Global Coordination and Challenges of Technical Standards…	2025	Source
120	Zincir, B.A.; Arslanoglu, Y. Comparative Life Cycle Assessment of Alternative Marine Fuels. Fuel 2024, 358,…	2024	Source
121	Global Industry Alliance to Support Low Carbon Shipping Alternative Marine Fuels: Regulatory Mapping; IMO…	2025	Source
122	European Maritime Safety Agency Study Investigating the Safety of Ammonia as Fuel on Ships --- Part 7:…	2025	Source
123	Hwang, I.; Jeong, B.; Jang, H.; Kim, D.; Jee, J. An Integrated Qualitative--Quantitative Risk Assessment for…	2025	Source
124	Yadav, A.; Jeong, B. Safety Evaluation of Using Ammonia as Marine Fuel by Analysing Gas Dispersion in a Ship…	2022	Source
125	Duong, P.A.; Kim, H.J.; Ryu, B.R.; Kang, H. A Quantitative Risk Analysis during Truck-to-Ship Ammonia…	2024	Source
126	Lankahaluge, A.S.; Graham, T.; Wang, H.; Bashir, M.; Blanco-Davis, E.; Wang, J. Formal Safety Assessment for…	2025	Source
127	International Maritime Organization MSC.1/Circ.1212/Rev.2 - Revised Guidelines on Alternative Design and…	2024	Instrument
